# Glycative Stress Disrupts the Mitochondrial‐Lysosome Axis and Promotes Geroconversion in Aging Cardiomyocytes

**DOI:** 10.1111/acel.70444

**Published:** 2026-03-13

**Authors:** Diana Bou‐Teen, Simonas Valiuska, Elisabet Miro‐Casas, Chiara Rubeo, Elena Bonzon‐Kulichenko, Zuzana Nichtova, Celia Fernandez‐Sanz, Javier Inserte, Antonio Rodriguez‐Sinovas, Begoña Benito, Eduard Ródenas‐Alesina, Jesús Vázquez, Ignacio Ferreira‐González, Marisol Ruiz‐Meana

**Affiliations:** ^1^ Cardiovascular Diseases Research Group Vall d'Hebron Institut de Recerca (VHIR), Vall d'Hebron Hospital Universitari, Vall d'Hebron Barcelona Hospital Campus Barcelona Spain; ^2^ Centro de Investigación Biomédica en Red de Enfermedades Cardiovasculares (CIBER‐CV) Madrid Spain; ^3^ Department of Clinical and Biological Sciences University of Torino Torino Italy; ^4^ DOE Research Group Institute of Biomedicine of the University of Castilla‐La Mancha (IB‐UCLM) Albacete Spain; ^5^ MitoCare Center for Mitochondrial Imaging Research and Diagnostics, Department of Pathology Anatomy & Cell Biol., Thomas Jefferson University Philadelphia Pennsylvania USA; ^6^ Center for Translational Medicine, Sidney Kimmel Medical College, Thomas Jefferson University Philadelphia Pennsylvania USA; ^7^ Cardiovascular Proteomics Laboratory Centro Nacional de Investigaciones Cardiovasculares Carlos III Madrid Spain; ^8^ Centro de Investigación Biomédica en Red de Epidemiología y Salud Pública (CIBER‐ESP) Madrid Spain

**Keywords:** AGEs, aging, cardiomyocytes, lipofuscin, methylglyoxal, mitochondria, senescence

## Abstract

Aging is a major risk factor for heart failure, yet the molecular mechanisms linking cardiac aging to the inflammatory pathophysiology of heart failure remain elusive. Mitochondrial dysfunction and defective organelle quality control are emerging hallmarks of the aging heart, but their biochemical underpinnings are poorly defined. Using comprehensive glycomics, we found that cardiac mitochondria from physiologically aged mice (≥ 20 months) are the major intracellular reservoirs of advanced glycation end products (AGEs), derived primarily from the chemical attack of some α‐oxoaldehydes on proteins. This was associated with mild mitochondrial dysfunction and structural remodeling. Lysosomes in aged hearts were enlarged, more abundant, less acidic, and frequently loaded with lipofuscin. Notably, ~7% of cardiomyocytes showed proinflammatory senescence traits. In vitro, glycative stress in H9c2 myoblasts reproduced mitochondrial AGE buildup, dysfunction, and activation of the mitochondria–lysosome axis. However, AGE‐modified mitochondria impaired lysosomal acidification and proteolysis, hindering mitophagic clearance and contributing to lipofuscin accumulation. This sequence of events ultimately led to proinflammatory senescence in a subset of cells. These findings identify mitochondrial AGE accumulation as a novel mechanism of sublethal nonsolved aging‐associated stress that eventually triggers geroconversion in cardiomyocytes. This mechanism could facilitate the transition of the aging heart towards a failing phenotype.

## Graphical abstract

Cardiac aging promotes mitochondrial AGE accumulation driven by defective glycative stress detoxification. AGE‐modified mitochondria disrupt lysosomal proteolysis, impairing mitophagy and promoting lipofuscin buildup. This mechanism sustains chronic unresolved mitochondrial damage, driving proinflammatory senescence in cardiomyocytes and defining a maladaptive aging response that can determine the transition between cardiac health and the onset of heart failure.

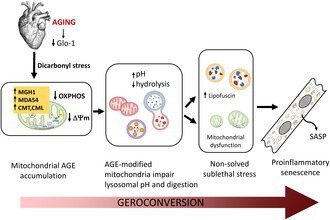

## 1 Introduction

Alterations in cellular metabolism is an important source of random molecular damage thoughout life (Avanesov et al. 2014; Breitenbach et al. 2014; Gladyshev et al. 2021), the deleterious consequences of which manifest when the escalating loss of molecular fidelity eventually exceeds the cell's repairing capacity (Hayflick 1998, 2007). Indeed, the accumulation of molecular damage is the cornerstone of aging (Gladyshev 2016; Gladyshev et al. 2021; Hayflick 1998) and it increases the organism's vulnerability to disease and death. This is particularly relevant for postmitotic long‐lived cells, like cardiomyocytes and neurons, in which molecular damage generated by their high metabolic activity cannot be diluted through cell division (Terman et al. 2008). As a result, cardiomyocytes and neurons accumulate indigestible lysosomal pigments during aging that are proposed to be risk factors for heart failure (Ahmed et al. 2010) and neurodegenerative diseases (Wellings et al. 2017). A paradigm of molecular damage derived from metabolism is the stochastic chemical attack of dicarbonyl compounds to biological macromolecules (Rabbani and Thornalley 2015). Dicarbonyl stress encompasses a broad range of reactive carbonyl species generated through distinct biochemical pathways. Among these, α‐oxoaldehydes (such as methylglyoxal [MGO] and glyoxal [GO]) originate mainly from glucose metabolism and are key mediators of glycative stress, whereas lipid‐derived dicarbonyls (including 4‐hydroxynonenal [HNE], malondialdehyde [MDA], 4‐oxo‐2‐nonenal [ONE], and isolevuglandins) originate from lipid peroxidation and represent a different form of carbonyl toxicity (Davies et al. 2020; Tao et al. 2020). Glycative stress results from the accumulation of α‐oxoaldehydes that, if not efficiently detoxified, react with positively charged amino acid residues in proteins to form heterogeneous and often irreversible adducts collectively known as advanced glycation end‐products (AGEs) (Brownlee 1995; Rabbani and Thornalley 2015). The pathogenic impact of AGEs depends on their abundance, location and ability to interfere with protein's structure and solubility, and on their chemical nature that may render them resistant to proteolysis (Grimm et al. 2010).

The high cytotoxicity of glycative stress is counteracted by the glyoxalase enzymes (Glo‐1 and Glo‐2) that rapidly convert MGO and GO into non‐toxic D‐lactate (Thornalley 1993). The Glo system is the universal detoxification mechanism shared by all eukaryotic cells to prevent glycative stress, that is, the abnormal accumulation of glucose‐derived dicarbonyl compounds that thermodynamically favors the generation of AGEs (Rabbani and Thornalley 2015). Glycative stress has been documented in some of the most prevalent aging‐associated diseases (Kuhla et al. 2007; Prinsen et al. 2020; Rabbani and Thornalley 2015; Richarme et al. 2015; Vitek et al. 1994; Williams et al. 2011) but its contribution to the onset and progression of these diseases remains unclear (Vašková et al. 2025). In the aging heart, we demonstrated a deficiency in Glo‐dependent detoxification that is shared by humans (≥ 75 years) and mice (≥ 20 months) (Ruiz‐Meana et al. 2019), despite a dramatic species‐dependent lifespan difference. Deficient Glo activity favors spontaneous AGE generation in cardiomyocytes during aging, being ryanodine receptor at the sarcoplasmic reticulum (Ruiz‐Meana et al. 2019) and FoF1‐ATP synthase in mitochondria (Bou‐Teen et al. 2022) two functionally relevant targets of glycative damage.

Aging is the main independent risk factor for the development of heart failure with preserved ejection fraction (HFpEF), a prevalent inflammatory disease with an ominous prognosis that causes a high rate of disability, loss of quality of life and death. To date, its complex pathophysiology remains poorly understood and there are no effective treatments capable of preventing its progression or reducing its mortality (Abdellatif and Kroemer 2022). The aging heart displays structural and metabolic changes strongly resembling those of failing hearts (North and Sinclair 2012; Strait and Lakatta 2012), which could explain why the incidence of HFpEF doubles with each decade of life after age 50 (Abdellatif and Kroemer 2022; North and Sinclair 2012). The aim of our study was to investigate whether the glycative stress endogenously present in the aging heart can eventually trigger senescence in cardiomyocytes, a mechanism that could drive the transition of a healthy heart towards an inflammatory failing phenotype. By using mass spectrometry combined with high resolution images and functional studies, we identified mitochondria as a main reservoir of intracellular AGEs in the aging heart, and established a cause‐effect relationship between sustained mitochondrial glycative damage and cardiomyocyte senescence mediated by lysosome alkalinization and failure to digest defective AGE‐containing mitochondria. This mechanism could perpetuate mitochondrial energy deficiency during aging and contribute to the onset of aging‐associated HFpEF. Its identification is expected to open new therapeutic opportunities.

## 2 Methods

### 2.1 Mouse Myocardium

Myocardium and cardiac myocytes were obtained from young (4–6 months) and old (≥ 20 months) male C57BL/6J mice (150 mg/Kg pentobarbital i.p.). All procedures involving animal tissue were approved by the ethical committee of the Vall d'Hebron Research Institute (CEEA 53/20) and were conducted in accordance with the EU directive 2010/63EU and Spanish disposition RD 53/2013 on protection of animals for scientific purposes.

#### 2.1.1 Heart Preparation and Cryopreservation With Sucrose for Frozen Tissue Sections

Heart fixation was achieved by the infusion of 4% paraformaldehyde (PFA) in phosphate buffer using a 27‐G needle through an apex puncture and subsequent tissue immersion in the same fixative for an additional 20 h. Next, the heart was immersed in 15% sucrose in phosphate buffered saline (PBS) for 5 h at 4°C, and then 30% sucrose in PBS overnight prior to cryopreservation. Subsequently, the heart was embedded in OCT, immediately frozen in cold isopentane (−40°C), and stored at −80°C until use. Cryosections were obtained at 10 μm, left 30 min at room temperature (RT), and hydrated with PBS for 4 h at RT.

#### 2.1.2 p16^INK4a^ Immunofluorescence

For p16^INK4a^ immunostaining, hydrated cryosections from mouse myocardium were permeabilized with 0.5% saponin (47,036, Sigma) in blocking buffer (5% bovine serum albumin [BSA]‐PBS) for 15 min at RT. The primary antibody (p16^INK4a^ sc1661, SantaCruz; 4 μg) was conjugated with Alexa 568 using the ApexTM Alexa Fluor 568 antibody labelling Kit (A10494, Invitrogen), diluted in blocking buffer at 2 μg/mL and incubated overnight at 4°C. Nuclei were stained with 5 μg/mL Hoescht 33342 in PBS and slices were stored in Prolong Gold antifade mounting reagent (P10144, Invitrogen). A central Z‐plane image was acquired with a confocal microscope (Zeiss LS980) at 40× magnification.

#### 2.1.3 Wheat Germ Agglutinin Staining

To quantify the cross‐sectional area of cardiomyocytes, heart cryosections were incubated with 10 μg/mL WGA‐FITC in PBS (L4895, Sigma) for 1 h at RT to stain the sarcolemma of the cells. Nuclei were stained with 5 μg/mL Hoescht 33,342 and slices were stored in Prolong Gold antifade mounting reagent (P10144, Invitrogen). A central Z‐plane image was acquired with a confocal microscope (Zeiss LS980) at 40× magnification and the cardiomyocyte area was quantified with ImageJ software.

#### 2.1.4 Senescence Associated‐β‐Galactosidase Assay

Senescence associated β‐galactosidase (SA‐β‐Gal) activity was assessed at pH 6 as described (Anderson et al. 2019). Briefly, hydrated cryosections from mouse myocardium were incubated with citrate buffer at pH 6 (in mmol/L: 150 NaCl, 2 MgCl_2_, 40 citric acid, 12 sodium phosphate) supplemented with 5 mmol/L potassium ferricyanide (P8131, Sigma), 5 mmol/L potassium ferrocyanide (P3289, Sigma), and 2 mg/mL XGal substrate (5‐Bromo‐4‐chloro‐3‐indolyl β‐D‐galactopyranoside B4252, Sigma). After overnight incubation at 37°C, the cryosections were washed with PBS and bright field images were acquired with a Nikon Eclipse Ts2R microscope at 20X magnification.

#### 2.1.5 Quantification SASP Components by Quantitative RT‐PCR

Total RNA was isolated from myocardial tissue of young and aged mouse hearts using a commercial RNA extraction kit, and RNA concentration and purity were assessed spectrophotometrically. Reverse transcription was performed using a standard high‐capacity cDNA synthesis kit. Gene expression of SASP components, including *IL‐1a*, *IL‐1b*, *IL‐6*, *Ccl‐2*, *Edn3*, *Tnfsf11*, *Mmp9*, and *Tgfb2*, was quantified by real‐time quantitative PCR using TaqMan probes. Reactions were run on a real‐time PCR system according to the manufacturer's instructions. Gene expression levels were normalized to *Gapdh* and expressed as 2‐fold change with respect to baseline (2ΔCt).

#### 2.1.6 Mass Spectrometry Analysis, and Modified Peptide and Protein Identification and Quantification

Mouse myocardium homogenates were analyzed by nano‐liquid chromatography–tandem mass spectrometry (nanoLC‐MS/MS) as described previously (Bou‐Teen et al. 2022) (see Supporting Information; “Supplementary Methods”). Quantification of modified peptides and proteins was performed using the Generic Integration Algorithm on the basis of the WSPP model (García‐Marqués et al. 2016) and changes in abundance were expressed in standardized units corrected by the corresponding protein abundance (Zpq). Differences between the distributions of Zpq were analyzed by the two‐tailed Kolmogorov–Smirnov test.

#### 2.1.7 Transmission Electron Microscopy

Mouse hearts were perfused in a Langendorff system with calcium Tyrode solution and fixed as described previously (Bou‐Teen et al. 2022). Longitudinal, ultrathin sections (65–80 nm) were obtained with a diamond knife (Diatome‐US, USA) and caught on a copper grid covered with formvar film. Images were obtained with an FEI Tecnai 12 TEM fitted with an AMT XR‐111 10.5 Mpx CCD camera at 3200–15,000× magnification (80 kV).

### 2.2 Isolated Mouse Cardiomyocytes

Calcium tolerant rod‐shaped cardiomyocytes were isolated by Langendorff perfusion as previously described (Fernandez‐Sanz et al. 2014) and plated on laminin‐coated glass WillCo dishes or processed for PCR.

#### 2.2.1 Mitochondrial Membrane Potential in Cardiomyocytes

Isolated cardiomyocytes were incubated with 100 nmol/L TMRE (T669, Invitrogen) in HEPES buffer (mmol/L: 140 NaCl, 3.6 KCl, 1.2 Mg_2_Cl, 1 CaCl_2_, 20 HEPES pH 7.4, 5 glucose) for 25 min at 37°C, washed and post‐incubated. Cell images were obtained at 575 nm using a spectral confocal microscope (Zeiss Laser Scanning Microscope (LSM) 980, 60×). Maximal mitochondrial membrane depolarization was achieved by the addition of 200 μmol/L dinitrophenol (DNP). Fluorescence mean values were quantified with ImageJ software and expressed as arbitrary units of fluorescence (a.u.f.).

#### 2.2.2 Mitochondrial and Lysosomal Abundance and Spatial Inter‐Organelle Interaction in Cardiomyocytes

To quantify mitochondrial density and lysosome number and size in intact cells, live cardiomyocytes were loaded with 50 nmol/L MitoTracker Red FM (MTR, M22425 Invitrogen) for 30 min at 37°C, or with 50 nmol/L LysoTracker Green DND‐26 (LTG, L7526 Invitrogen) for 5 min at 37°C, and visualized at 650 and 510 nm, respectively, with Zeiss LSM980 (60×). Morphometric analyses were done in background‐subtracted cell images using ImageJ software. Mitochondria‐lysosome anatomical proximity was determined from Mander's coefficient (M2) after simultaneous staining with MTR and LTG using the Colocalization plugin (ImageJ software).

#### 2.2.3 Lysosomal pH in Cardiomyocytes

Lysosomal pH was measured in cardiomyocytes from young and old mice by a double staining with the pH‐sensitive LysoSensor Green DND‐189 (LSG, 100 nmol/L, Invitrogen L7535) and the pH‐insensitive LysoTracker Red DND99 (LTR, 100 nmol/L, Invitrogen L7528), 30 min at 37°C, as previously described (Sun et al. 2020). Images were acquired at 502 and 590 nm with Zeiss LSM980 (40×). Fluorescence mean values were determined using ImageJ software, and the LSG/LTR ratio was calculated for qualitative estimation of lysosomal acidity.

#### 2.2.4 Lipofuscin Pigment

To identify intracellular lipofuscin pigment, intact cardiomyocytes with nuclei stained with 5 μg/mL Hoescht 33342 were visualized at 500–587 nm range of spectral emission autofluorescence (488 nm excitation) using Zeiss LSM980 (60X). Mean autofluorescence was analyzed with ImageJ software in background‐subtracted images and expressed as number of particles per cell. In TEM images, lipofuscin pigment was identified as electrodense aggregates with an irregular surface in the myocardium of old mice. This type of structure was absent in the myocardium of young mice.

#### 2.2.5 Quantification of Senescence Markers by Quantitative RT‐PCR in Cardiomyocytes

Total RNA was isolated from cardiomyocytes purified from young and aged mouse hearts, and reverse transcription was performed using a standard cDNA synthesis protocol. Gene expression of canonical markers of cellular senescence, including *Cdkn2a* (p16^INK4a^), *Cdkn2b* (p15^INK4b^), and *Cdkn1a* (p21^Cip1^), was quantified by real‐time quantitative PCR using TaqMan probes. Gene expression levels were normalized to *Gapdh* and expressed as relative fold change using the ΔCt method.

### 2.3 Isolated Mitochondria

Subsarcolemmal (SSM) and interfibrillar (IFM) heart mitochondria were obtained from hearts of young and old mice by differential centrifugation. The isolation protocol used here has been previously characterized for its mitochondrial enrichment and functional integrity (Fernandez‐Sanz et al. 2014).

#### 2.3.1 Analysis of Mitochondrial Oxygen Consumption

Oxygen consumption rates were measured in SSM and IFM using a Clark‐type electrode (Oxygraph, Hansatech) in the presence of 2 mmol/L malate and 5 mmol/L glutamate (resting respiration, state 2) and after the stimulation with ADP (maximal oxygen consumption, state 3). Oxygen consumption rates are expressed as nmolO_2_/min × CS (citrate synthase) units. The respiratory control rate (RCR) was calculated as state 3/state 2 and was used as an index of respiration coupling. CS activity was determined colorimetrically and used both to quantify mitochondrial content and to normalize respiration data. For these functional assays, enriched mitochondrial fractions are sufficient, as minor contamination from other cellular compartments does not affect oxygen consumption or CS activity. Mitochondrial yield corresponds to total mitochondrial protein/mg of myocardial tissue.

#### 2.3.2 Western Blot of Glycated Proteins in Mitochondria

For Western blot analyses, isolated mitochondria underwent and additional purification step. SSM and IFM were further diluted in a buffered sucrose solution (mmol/L: 290 sucrose, 5 MOPS pH 7.2, 2 EGTA) containing 17% percoll and centrifuged at 12,500*g* 8 min 4°C. Fifty μg of mitochondrial protein were diluted in Laemmli buffer (S3401, Sigma), resolved on 10% acrylamide gels under denaturing conditions and subsequently transferred onto a nitrocellulose membrane. Primary antibodies (rabbit anti‐CML ab27684, Abcam 1:1000; mouse anti‐MAGE STA‐011, Cell Biolabs, 1:1000; mouse anti‐SDHA ab14715, Abcam 1:20000) were incubated overnight at 4°C in blocking buffer (Roti‐Block (A151.1, Roth) in TBS‐0.1% Tween). Secondary antibodies (anti‐rabbit‐HRP 31460 Pierce and anti‐mouse IgG‐HRP A9309 Sigma) were incubated for 1 h in blocking buffer at RT. Chemiluminescence (ECL Prime Western blotting detection reagent, Cytiva) was detected using the Odyssey Fc imaging system (Li‐Cor) and images were analyzed with ImageJ software.

### 2.4 Mitochondrial Phagocytosis and Lysosomal Digestion by Macrophage‐Like RAW 264.7 Cells

#### 2.4.1 Phagocytosis and Lysosomal Digestion

To investigate the effect of age‐dependent mitochondrial glycation on the efficiency of lysosomal digestion, cardiac mitochondria from young and old mouse hearts were co‐incubated with RAW 264.7 (ATCC, US), a murine macrophage‐like cell line with an active phagocytic capacity and an enzyme‐rich lysosomal compartment. Briefly, isolated mitochondria were labeled with 150 μmol/L of pHrodo deep red cell tracker (P35357, Thermofisher) for 1 h at RT in sucrose medium (in mmol/L, 290 sucrose, 10 MOPS, pH 7.4, 1 EGTA, with 1% BSA), a pH‐sensitive dye that becomes fluorescent under the acidic environment of lysosomes. Labeled mitochondria were centrifuged (5000*g*, 5 min) and subsequently added (in a concentration of 100 ng/μL) to RAW 264.7 cells previously seeded at 50.000 cells per well in a μ‐Slide 8 Well (80,826, Ibidi GmbH). Cytochalasin D (ab234622, Abcam, 1:100), a polymerization inhibitor, was added to a subset of macrophages 1 h prior to co‐incubation with mitochondria to inhibit phagocytosis (internal control of the probe specificity). Time‐lapse images (every 4 min) were acquired during 400 min at 37°C using Leica Thunder Wide Field Fluorescence Microscope (DMi8, Leica) (20×). The initial engulfment of mitochondria and subsequent lysosomal digestion by macrophages was monitored by the combination of bright field microscopy with the assessment of changes in 695 nm fluorescence intensity using ImageJ software.

#### 2.4.2 Lysosomal Size and pH

Vesicular pH and size were quantified in RAW 264.7 cells following co‐incubation with isolated cardiac mitochondria from young or old mice for 0 min (basal) or 400 min (7 h). Cells were loaded with LysoSensor Green (LSG, 100 nmol/L) and LysoTracker Red (LTR, 100 nmol/L) for 30 min at 37°C, and images were acquired at 40× using a Zeiss LSM980 (Ex 502 nm for LSG and 590 nm for LTR). Fluorescence intensity of individual vesicles, LSG/LTR ratios and lysosomal size were quantified using ImageJ software. For vesicular pH calculation, a calibration curve was generated in MES buffer (in mmol/L: 125 KCl, 25 NaCl, 25 MES) over a pH range of 4.5–6.5, using LSG (12 nmol/L) and LTR (14 nmol/L). Fluorescence was measured with a multimode plate reader (iD3, Molecular Devices), and LSG/LTR ratios were plotted against pH to calculate vesicular pH in experimental samples. The percentage of vesicles displaying impaired acidification (pH > 5.5.) was also calculated.

### 2.5 H9c2 Cells

#### 2.5.1 Cell Culture and Induction of Glycative Stress

H9c2 cells (ATCC, US) were grown in culture DMEM medium (30‐2002 ATCC) with 10% FBS for 24 h to promote cell attachment. To mimic the glycative stress present in aging, cells were cultured up to 9 days in starved medium (2% FBS) supplemented with 80 μmol/L methylglyoxal (MGO, M0252, Sigma) and 2 μmol/L S‐p‐bromobenzylglutathione cyclopentyl diester (SML1306, Sigma), a pharmacological reversible inhibitor of Glo‐1 (“MGO group”) or in starved DMEM (“Control group”). Where indicated, a positive control of senescence was included by the addition of doxorubicin (single dose of 0.1 μmol/L, 24 h) followed by the incubation in starved fresh medium (“DOXO group”). Unless otherwise indicated, the biochemical/morphometrical quantifications were performed in basal conditions, after 3 days of treatment and at the end of the experiment (Day 9).

#### 2.5.2 Glyoxalase Activity, Glutathione Levels and Viability in H9c2 Cells

To check whether the induction of glycative stress results in partial inhibition of Glo‐1 activity, as in the aging heart (Ruiz‐Meana et al. 2019), cells were lysed with MPER (78503, Thermofisher) after 24 h of Glo inhibition, and Glo‐1 activity was determined in cell extracts by monitoring S‐lactoylglutathione absorbance at 240 nm over time using a multimode reader (iD3, Molecular Devices), following an optimized procedure previously described (Arai et al. 2014). Glo‐1 activity was expressed as nmols S‐lactoylglutathione/min × mg protein. Total glutathione levels were also determined from cell extracts after 24 h of treatment using a standard curve and were expressed as nmols total GSH/mg protein (Ruiz‐Meana et al. 2019). Cell viability was quantified by the Trypan Blue exclusion test.

#### 2.5.3 Quantification of Glycated Proteins in Mitochondria Isolated From H9c2 Cells

The degree of mitochondrial glycation in response to chronic glycative stress was analyzed in mitochondria isolated from cultured H9c2 cells at Day 9. Cells were trypsinized and collected in cold isolation buffer (in mmol/L: 290 sucrose, 10 HEPES, pH 7.4, 1 EDTA) supplemented with 1 mmol/L PMSF, 10 μL/mL of protease inhibitor cocktail (P8340, Sigma) and 100 U DNase (D2502, Sigma), mechanically lysed (using a 1 mL syringe provided with a 27G needle). Mitochondria were then isolated from lysates by differential centrifugation (Bou‐Teen et al. 2022) and solubilized with 0.1% Triton X‐100. Fifty μg of protein were resolved in 10% polyacrylamide gels under denaturing conditions and transferred onto nitrocellulose membrane. Mouse anti‐MAGE antibody (STA‐011, 1:1000) was used to immunoblot. Chemiluminescence was detected with Odyssey Fc imaging system (Li‐Cor) and bands quantified with ImageJ software. Anti‐VDAC (abcam ab14734, 1:5000) was included as protein loading control.

#### 2.5.4 Changes in Mitochondrial Membrane Potential in H9c2 Cells

Mitochondrial membrane potential (ΔΨm) was measured in JC‐1 loaded H9c2 cells (5 μmol/L, 8 min, 37°C) at different time points of exposure to glycative stress (or control) by 488 nm excitation and simultaneous emission at 520 and 590 nm, using a Zeiss LSM980 (60X). Fluorescence mean values were quantified using ImageJ software and reduction in ΔΨm was detected as a decay in 590/520 emission ratio with respect to basal conditions. Maximal depolarization was achieved with 200 μmol/L DNP.

#### 2.5.5 Energetic Metabolism in H9c2 Cells

ATP production rate was monitored at different time points of glycative stress (or control) by real‐time ATP rate assay (103591‐100, Agilent) in a Seahorse XFp analyzer (Agilent Technologies, Seahorse Bioscience, USA). H9c2 cells were trypsinized and seeded at 15,000 cells per well coated with 0.1% gelatin in Seahorse XF HS Mini 8‐well plates (103022‐100, Agilent). ATP rate assay was performed in DMEM medium at pH 7.4 supplemented with substrates (in mmol/L: 25 glucose, 1 pyruvate and 2 L‐glutamine). Inhibitors of mitochondrial respiration (in μmol/L: 1.5 oligomycin, 0.5 rotenone and 0.5 antimycin A) were sequentially loaded in each well during the assay. At the end of the experiment, cells were lysed (0.1% Triton X‐100) and protein was determined by Bradford assay. Total ATP production rate (pmols/min × μg protein) was calculated using Wave software (v2.6.1., Agilent), as previously described (Bou‐Teen et al. 2022).

#### 2.5.6 Mitochondrial Abundance, Size and Branching in H9c2 Cells

Changes in mitochondrial abundance, size (perimeter), and branching in response to glycative stress were evaluated in H9c2 cells labeled with 50 nmol/L MTR (30 min, 37°C), as described for intact cardiomyocytes (see above). Mitochondrial perimeter (μm) and degree of interconnectivity/branching (number of mitochondrial junctions per μm^2^) were analyzed using the Skeletonize 3D/2D plugin with binary images (ImageJ software).

#### 2.5.7 Spatial Proximity Between Mitochondria and Lysosomes in H9c2 Cells

The dynamic changes of the interaction between lysosomes and mitochondria in response to glycative stress were analyzed by Mander's coefficient colocalization (M2) in H9c2 cells simultaneously loaded with 200 nmol/L MTR and 50 nmol/L LTG, as described for cardiomyocytes (see above).

#### 2.5.8 Functional Interaction Between Mitochondria and Lysosomes in H9c2 Cells

The degree of mitochondrial degradation and functional interaction between lysosomes and mitochondria were assessed in live H9c2 cells by triple staining with MitoBlue (MB, SCT086 Sigma), MTR and LTG, as previously described (Sánchez et al. 2020). Upon initial localization within mitochondria, MB is gradually transported out of the mitochondria (either as vesicles or as mitochondrial fragments), ending up in lysosomes, as part of the mitochondrial quality control process. To study the mitochondrial recycling activity in response to glycative stress, H9c2 cells at Day 0, 3 and 9 of each treatment group were loaded with 200 nmol/L MTR and 5 μmol/L MB for 30 min at 37°C in culture medium without FBS, and washed for 30 min in fresh culture medium. Prior to microscopic observation, cells were incubated with 50 nmol/L LTG for an additional 5 min. Images were obtained using LSM980 (60X) at 600 nm (red), 465 nm (blue) and 517 nm (green) and the analysis of Mander's coefficient (M2) of MB‐LTG (indicative of lysosomal mitophagy) and of MB‐MTR (indicative of intact mitochondria) was obtained from Colocalization plugin (ImageJ software).

#### 2.5.9 Lysosomal pH in H9c2 Cells

The impact of glycative stress on lysosomal pH was analyzed in H9c2 cells simultaneously loaded with LSG and LTR, as described for cardiomyocytes (see above).

#### 2.5.10 Hydrolytic Lysosomal Activity

The efficiency of lysosomal hydrolytic activity in response to glycative stress was measured in H9c2 cells using the Lysosomal Intracellular Activity Assay Kit (ab234622, Abcam). Briefly, cells were incubated in starved culture medium with 5 μg/mL of lysosome‐specific self‐quenched substrate (2 h at 37°C). The substrate is insensitive to pH and is taken up by cells as an endocytic cargo. Upon its degradation by lysosomal enzymes, it is de‐quenched and the resulting fluorescent signal, which is proportional to intracellular lysosomal activity, is analyzed by flow cytometry (LSR Fortessa, LSRII, BD). As an internal control of specificity, some cells were incubated with 5 μmol/L cytochalasin D to inhibit lysosome hydrolysis (data not shown). Subsequently, H9c2 cells from each treatment were loaded with 50 nmol/L LysoTracker Deep Red (LTDR, Thermofisher L12492) for 5 min at 37°C to estimate the lysosomal pool. Fluorescence at 530 nm (hydrolytic activity) and 670 nm (lysosomal pool) was analyzed using Flowjo software. Data on substrate digestion were normalized by lysosomal pool. SSC‐W versus SSC‐H plots were used to identify individual cells and discriminate between normal‐sized and enlarged (senescent) cells using predefined windows.

#### 2.5.11 Lipofuscin Autofluorescence in H9c2 Cells

Lipofuscin generation in response to glycative stress was detected by intracellular autofluorescence as described for cardiomyocytes.

#### 2.5.12 Senescence‐Associated β‐Galactosidase Activity and Cell Area in H9c2 Cells

For SA‐β‐galactosidase activity, cells from each treatment group were fixed with 4% PFA in PBS (10 min, RT) and subsequently incubated in 10 mmol/L citrate buffer pH 6 with 2 mg/mL X‐gal for 20 h at 37°C, as described for mouse myocardium (see above). The percentage of SA‐β‐galactosidase (+) cells and the cell area (μm^2^) were quantified using ImageJ software.

#### 2.5.13 Assessment of LC3B Processing in H9c2 Cells

LC3B processing and LC3B‐II accumulation in response to lysosomal inhibition, used as an indicator of autophagic flux, were assessed in cultivated H9c2 cells at 48 h of chronic dicarbonyl stress. Where indicated, cells were treated with the V‐ATPase inhibitor concanamycin A (10 μmol/L, 2 h) prior to collection. Cells were lysed in M‐PER Mammalian Protein Extraction Reagent supplemented with protease inhibitors. Equal amounts of protein (30 μg) were separated by SDS–PAGE under denaturing conditions and transferred onto PVDF membranes. Membranes were incubated with primary antibodies against LC3B (rabbit, 1:1000, Invitrogen) and vinculin (mouse, 1:10000, Sigma‐Aldrich), followed by HRP‐conjugated secondary antibodies (anti‐rabbit‐IgG‐HRP, 31460, Pierce and anti‐mouse IgGκ BP‐HRP sc‐516102, Santa Cruz Biotechnology). Chemiluminescent signals were detected and quantified using ImageJ software.

#### 2.5.14 p16^INK4a^ Immunofluorescence

For p16^INK4a^ immunostaining, cells were fixed with 4% PFA in PBS, permeabilized with 0.5% saponin and incubated with 2 μg/mL p16^INK4a^ antibody conjugated with Alexa 568 as described for cardiomyocytes. Images were acquired with LSM980 (40X). The percentage of p16^INK4a^ (+) cells was quantified using ImageJ software.

#### 2.5.15 Quantification of SASP Components by Quantitative RT‐PCR in H9c2 Cells

Gene expression of cytokines and chemoattractant factors of SASP was determined by RT‐PCR from RNA extracts of H9c2 cells in each treatment group. Primers used were Rn01410330_m1 (*IL‐6*), Rn00578225_m1 (*Cxc11*), Rn00676060_m1 (*TGF‐β2*), and Rn01755284_m1 (*Edn3*) (Thermofisher). Gene expression was normalized against *GAPDH* (Rn01775763_g1) and expressed as 2‐fold change with respect to baseline (2ΔCt).

### 2.6 Statistical Analysis

Data are expressed as mean ± standard error of the mean (SEM). A two‐tailed *t*‐test for independent or paired samples was applied to data following a normal distribution. The non‐parametric Mann–Whitney test for medians was applied for data not following a normal distribution. ANOVA analysis was used for comparisons between more than two groups. Differences of *p* ≤ 0.05 were considered as statistically significant. Statistical analyses were performed with SPSS v.20 software (New York, USA).

## 3 Results

### 3.1 Mitochondria Are the Main Intracellular Targets of Glycative Stress in the Aging Heart

**FIGURE 1:**
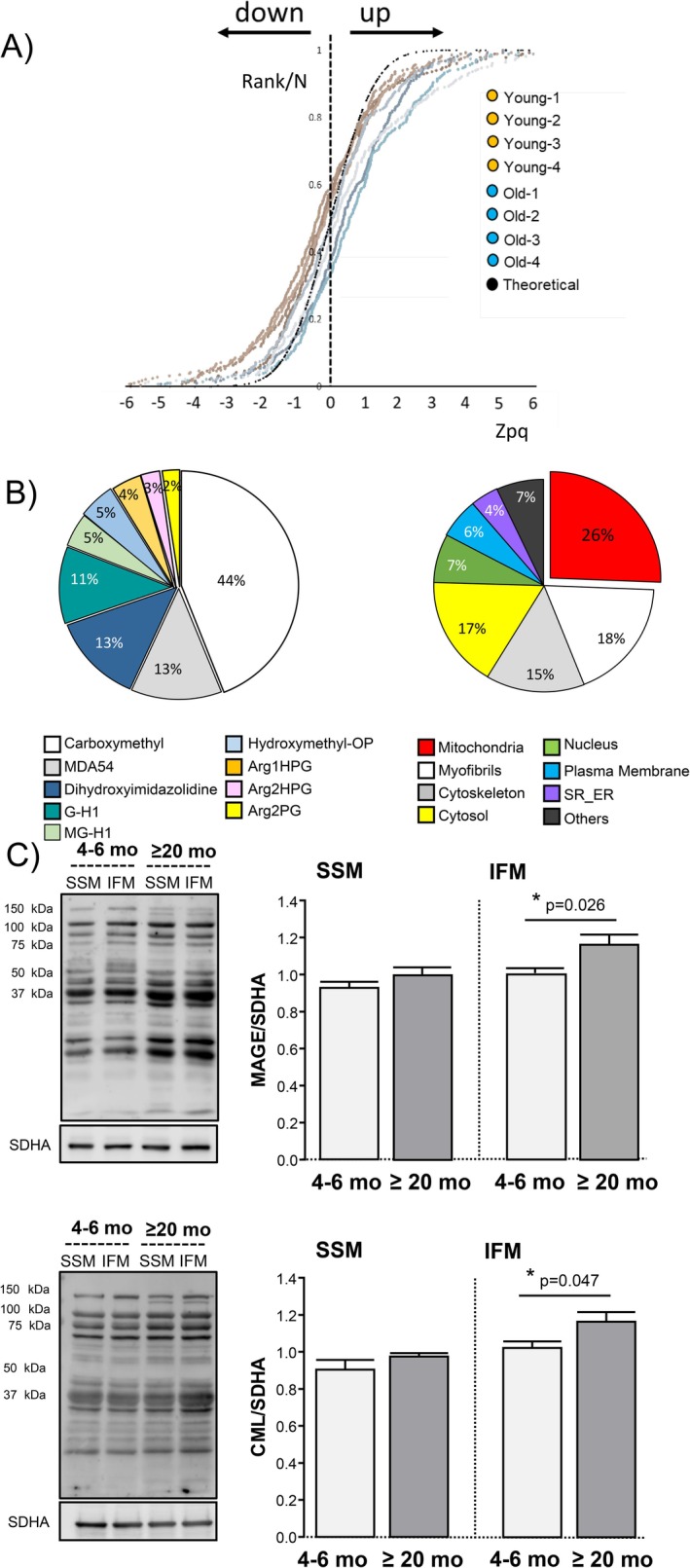
Aging increases the abundance of AGE‐modified proteins in the heart, which preferentially accumulate in mitochondria. (A) Sigmoid curves represent the cumulative frequency distribution of the global AGE‐modified peptides (Zpq values) in the myocardium of old (blue) versus young (orange) mice detected by quantitative mass spectrometry, for all peptides containing any type of AGE; a displacement towards the right indicates an increased concentration. Aging increases the abundance of AGE‐modified peptides in the heart. The black sigmoid is the theoretical null hypothesis (standard distribution). (B) Pie chart depicting the abundance (in percentage) of the 9 different chemical types of AGEs identified in the proteins of mouse myocardial homogenates (left) and their intracellular distribution (right). Highlighted in red is the percentage of AGEs localized in the mitochondria. Data correspond to *n* = 4 mice per group. (C) Western blots of subsarcolemmal (SSM) and interfibrillar (IFM) mitochondria isolated from young and old mouse hearts immunolabeled with anti‐MGO‐derived AGEs (MAGEs, upper panel) and anti‐CML (lower panel). Bar graphs represent the quantification of the optical density (OD), expressed with respect to the loading control succinate dehydrogenase, subunit A (SDHA). Data correspond to mean ± SEM of *n* = 5 mice per group.

Nano‐liquid chromatography–tandem mass spectrometry identified a total of 32684 peptides in the murine heart, 2185 of which (corresponding to 645 proteins) showed some type of glycation. The differential analysis of the chemical adducts documented 9 different types of AGEs, the relative abundance of which was significantly increased in the heart of mice ≥ 20 months with respect to young adults (4–6 months), as disclosed by frequency distribution analysis (Figure 1A). Arginine was the main target of glycation during aging, determined by the increased amount of 1HPG, 2HPG, MG‐H1, dihydroxyimidazolidine, G‐H1, and MDA54; and carboxymethyl in lysine (82% of total carboxymethyl) and tryptophan (18% of total carboxymethyl) was the second most abundant AGE (Figure 1B). Quantification of the intracellular location of glycated peptides indicated that mitochondria concentrate the largest fraction of glycation (up to 26% of total AGEs) (Figure 1B). Myofibrillar peptides represented the second most affected compartment (18%), followed by cytosolic (17%) and cytoskeletal (15%) peptides. Despite the high chemical heterogeneity, the most abundant mitochondrial AGEs were MGH‐1, Arg1HPG, and carboxymethyl. The chemical nature of these compounds indicates that the main source of mitochondrial glycation comes from MGO (Perrone et al. 2020).

The contribution of aging to the glycation of mitochondrial peptides/proteins was corroborated by Western blot quantification of two different AGEs, that is, MGO‐induced AGEs (MAGE) and carboxymethyl‐lysine (CML), using highly purified heart mitochondria. The data show an increased burden of both types of AGEs in cardiac mitochondria of aging hearts with respect to young ones (Figure 1C). Specifically, interfibrillar mitochondria (IFM), in close contact with myofibrils (the second hub of intracellular AGEs) are the mitochondria that display higher levels of AGEs in the aging heart, whereas glycation of subsarcolemmal mitochondria (SSM) is not increased in aging (Figure 1C).

### 3.2 Aging‐Associated Changes in Heart Mitochondria and Lysosomes

**FIGURE 2:**
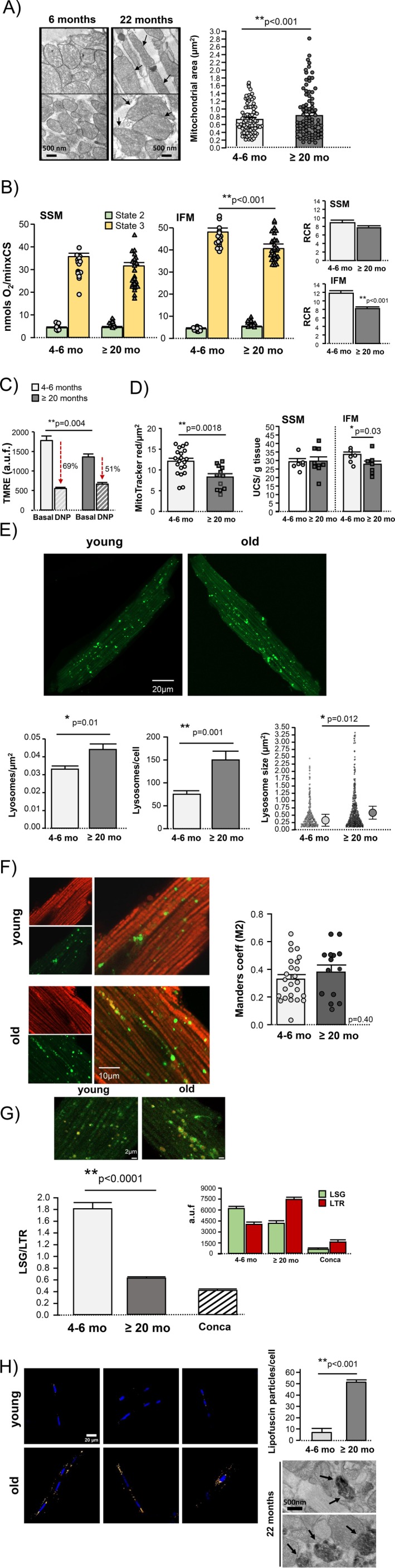
Structural and functional changes in cardiac mitochondria and lysosomes during aging. (A) High‐magnification (15,000×) TEM images of a field of mouse myocardium, two from a young mouse (6 months) and two from an old mouse (22 months), illustrating mitochondrial shape and size. Arrows indicate abnormally large or elongated individual mitochondria associated with aging. Right graph shows mitochondrial average area (μm^2^) quantified in TEM images of intact myocardium in both groups of age. Data are expressed as mean ± SEM of 80–90 mitochondria from 3 mice per group. (B) Oxygen consumption driven by complex 1 substrates (malate/glutamate) under resting conditions (state 2, green bars) and after maximal stimulation with ADP (state 3, yellow bars), measured in isolated subsarcolemmal (SSM) and interfibrillar (IFM) mitochondria from young and old mice using oximetry. The respiratory control rate (RCR) corresponds to state 3/state 2. Aging specifically affects IFM. Data are expressed as mean ± SEM for *n* = 10–12 mice per group, with 1–2 biological replicates per determination, and are normalized to citrate synthase (CS) activity (nmolO_2_/min*CS). (C) TMRE fluorescence as an indicator of mitochondrial membrane potential (ΔΨm) in cardiomyocytes from young and old mice, under resting conditions and after induction of maximal depolarization (200 μmol/L DNP) in each age group. Data correspond to mean ± SEM of 14–20 cardiomyocytes per group, from *n* = 3 mice per group. (D Effect of aging on heart mitochondrial pool, as quantified by 2 independent measurements: left) MitoTracker Red (+) particles per μm^2^ in isolated cardiomyocytes (mean ± SEM, of 15–22 cardiomyocytes per group, from *n* = 3 mice per group); (b) units of mitochondrial citrate synthase activity (UCS) per gram of tissue (mean ± SEM, *n* = 10–12 experiments per group). (E) Confocal images (60X) of intact cardiomyocytes from young and old mice labeled with LysoTracker Green to identify lysosomes. Bar graphs show the number of lysosomes per μm^2^ (left), the number of lysosomes per cell (middle), and the area of individual lysosomes (right) in cardiomyocytes from young (4–6 months) versus old mice (≥ 20 months). Data are presented as mean ± SEM, with *n* = 12–20 cardiomyocytes per group from *n* = 3 mice per group. (F) Confocal images (60×) of intact young and old mouse cardiomyocytes simultaneously labeled with MitoTracker Red (for mitochondria) and LysoTracker Green (for lysosomes) showing the fluorescent pattern for each marker, and the corresponding merge images. Right graph represents the Mander's overlap coefficient (colocalizing LysoTracker Green pixels with MitoTracker Red pixels with respect to total LysoTracker Green pixels). Data correspond to mean ± SEM of *n* = 13–22 cardiomyocytes per group from *n* = 3 mice per group. (G) Confocal images (60×) of intact young and old mouse cardiomyocytes labeled with LysoSensor Green (LSG) and LysoTracker Red (LTR). The reduction in LSG/LTR emission fluorescence corresponds to intralysosomal alkalinization. Positive control of lysosomal alkalinization was achieved by the addition of concanamycin A (10 nmol/L). Inset graph shows the independent fluorescence emission for each channel. Bar graph corresponds to mean ± SEM of *n* = 15–20 cells/group of *n* = 4 mice per group. (H) Confocal images (40×) showing lipofuscin auto‐fluorescence in young (4–6 months) and old (≥ 20 months) cardiomyocytes (3 examples per group). Nuclei are counterstained in blue with Hoescht. The upper right graph shows the average number of lipofuscin particles per cardiomyocyte (mean ± SEM of *n* = 30 cardiomyocytes per group of *n* = 3 mice per group). In the bottom right, high magnification (15,000×) TEM images of the myocardium of an old mouse with lipofuscin accumulation in lysosomes (arrows).

Ultrastructural analysis of intact myocardium by TEM showed an increased mitochondrial size in the cardiomyocytes of aging mice with respect to young ones due to the presence of abnormally enlarged mitochondria coexisting with normal‐sized mitochondria (Figure 2A). A small proportion of mitochondria (< 5%) in aged mice displayed various forms of aberrant cristae organization indicative of pathological alterations in mitochondrial architecture (Figure S1), in addition to the previously described onion‐like cristae morphology (Bou‐Teen et al. 2022). These abnormalities were not observed in young mice. Cardiac mitochondria isolated from aging mice displayed a decreased aerobic capacity (detected as complex‐1 mediated oxygen consumption) that specifically affected IFM population, whereas respiration of SSM was not modified by age (Figure 2B). Impaired mitochondrial O_2_ consumption during aging was manifested under maximal energy demand (i.e., in the presence of ADP or state 3) and resulted in slight respiratory uncoupling in IFM, indicated by depressed respiratory control rate (state 3/state 2), which remained preserved in SSM (Figure 2B). Under resting conditions, mitochondrial membrane potential (ΔΨm) was slightly decreased in cardiomyocytes from aging mice with respect to young ones, as quantified by DNP‐sensitive TMRE fluorescence (Figure 2C). In addition to the decreased respiratory efficiency per mitochondrial particle, myocardial aerobic capacity in the aging heart is further compromised by a reduction of total mitochondrial pool, as indicated by less amount of MitoTracker Red (+) mitochondria per cell surface in intact cardiomyocytes, and by a lower activity of citrate synthase (CS) in isolated mitochondria (Figure 2D). Such a reduction in mitochondrial mass in the aging heart occurs at the expense of IFM population, whereas the SSM population remains unchanged (Figure 2D). This is consistent with the mitochondrial yield data (i.e., mitochondrial protein relative to total cardiac protein), which also indicate a specific age‐dependent reduction in IFM (13.3 ± 1.0 mg/g vs. 9.03 ± 0.9 mg/g in young and old mice, respectively, *p* = 0.007), with no significant changes in SSM (9.0 ± 0.7 mg/g vs. 9.03 ± 0.9 mg/g in young and old mice, respectively, p = ns).

Since the lysosomal system is the primary mechanism for eliminating defective mitochondria, we investigated changes in cardiac lysosomal size and function during aging. Lysosomes in cardiomyocytes typically appeared as spherical (round or elliptical) organelles, regardless of age. However, the size and number of lysosomes were significantly increased in cardiomyocytes from aged mice compared with young ones, as quantified in confocal images of live cardiomyocytes stained with LysoTracker Green (Figure 2E). The increased average size was due to the presence of abnormally large‐sized lysosomes that coexist with normal‐sized lysosomes in certain cardiomyocytes from aged individuals. Despite the higher load of mitochondria damage in the aging heart (increased mitochondrial glycation and dysfunction, Figures 1 and 2B,C), the degree of lysosome interaction with mitochondria is not overactivated in cardiomyocytes from aged individuals, as indicated by a similar Mander's colocalization coefficient between LysoTracker Green and MitoTracker Red in cardiomyocytes from young and old individuals (Figure 2F). Nevertheless, the luminal pH was less acidic in the lysosomes of aging mice, as determined by the pH‐sensitive LysoSensor Green fluorescence in intact cardiomyocytes (Figure 2G), and cells exhibited a significant accumulation of lipofuscin pigment that was virtually absent in cardiomyocytes from young mice (Figure 2H). Lipofuscin granules correspond to non‐digested molecular aggregates containing cross‐linked and oxidized material, rich in AGEs (Yin 1996) that could also be observed in TEM images of myocardium of aged individuals as electrodense granular bodies, mainly located at the intralysosomal compartment (Figure 2H). Taken together, these data support the concept that lysosomes from aged cardiomyocytes cannot successfully digest intracellular AGEs, which tend to accumulate in the IFM, the mitochondrial population primarily affected by aging.

### 3.3 In the Aging Heart, Some Cardiomyocytes Exhibit a Senescent Phenotype

**FIGURE 3:**
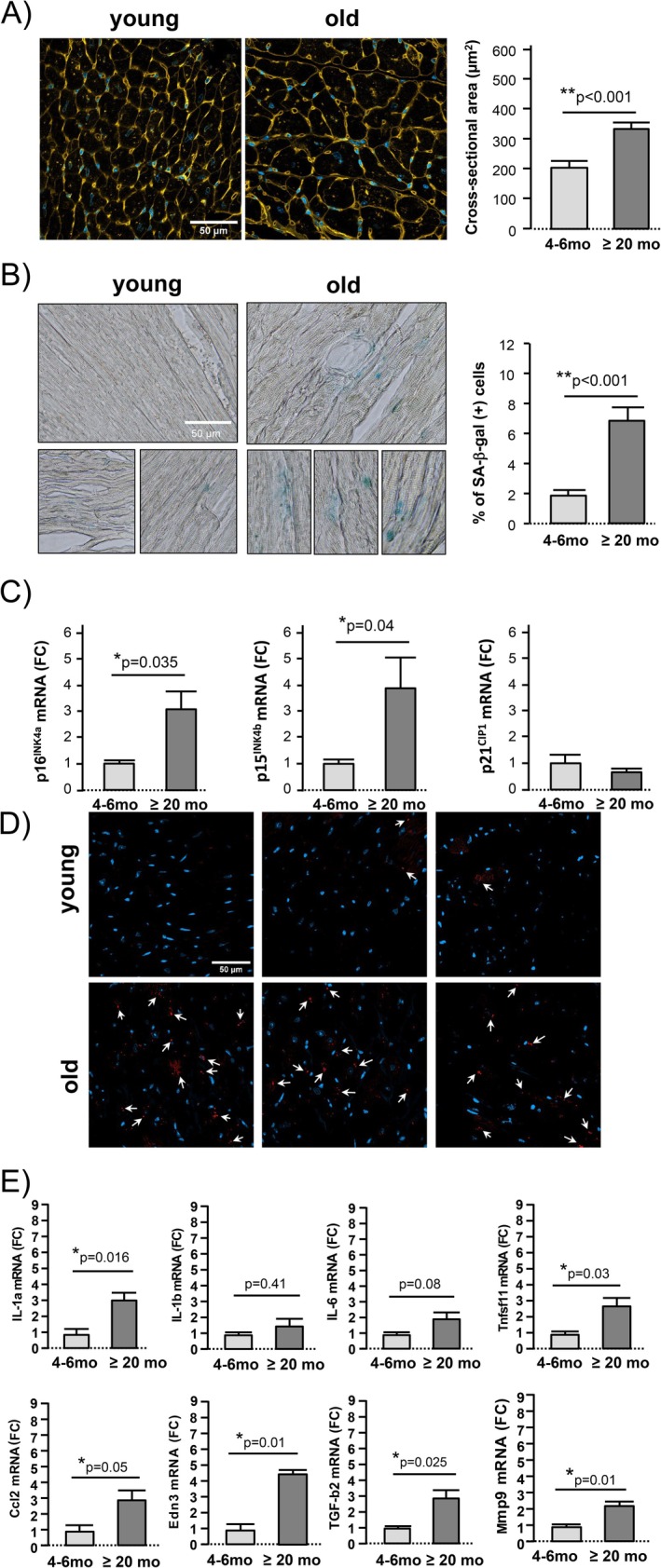
In the aging heart, some cardiomyocytes undergo senescent transformation. (A) Confocal images (40×) of myocardial cryosections from young and old mice in which the sarcolemma was stained with wheat germ agglutinin (WGA) to define cardiomyocyte perimeter. Nuclei are counterstained with Hoescht. The bar graph corresponds to the mean ± SEM cross‐sectional area of *n* = 25–40 cardiomyocytes per group of *n* = 2 mice per group. (B) Histological images (20X) of SA‐β‐Gal activity in myocardial cryosections (left), and the corresponding quantification of SA‐β‐Gal (+) cells (in blue) indicative of senescence (right). Data represent mean ± SEM of *n* = 3 mice per group. (C) Expression levels detected by PCR of senescent markers p16^INK4a^, p15^INK4b^, and p21^CIP1^ in isolated cardiomyocytes obtained from young and old mice. The graphs represent the mean ± SEM of the fold change in gene expression calculated with respect to the young mice from *n* = 4 mice per group (3 replicates per animal). (D) Confocal images (40×) of p16^INK4a^ immunostaining in myocardial cryosections from young and old mice (3 examples per group). Nuclei are counterstained with Hoescht (in blue). Arrows point to P16^INK4a^ (+) cells (in red) indicative of senescence. (E) Relative expression of SASP genes in myocardial tissue from young and aged mice, as determined by quantitative RT–PCR. The analyzed panel includes markers representative of pro‐inflammatory signaling, immune cell recruitment, extracellular matrix remodeling and fibrosis, as well as vasoactive and non‐canonical SASP components. Data are expressed as fold change relative to young mice and represent mean ± SEM from *n* = 3 mice per group (3 technical replicates per animal).

Although clinically asymptomatic, aged mice exhibit cardiac hypertrophy, with heart weight increasing from 125 ± 10 mg in young mice to 190 ± 11 mg in older mice (*p* = 0.001). At the cellular level, aging is associated with a significant increase in the average cross‐sectional area of cardiomyocytes (Figure 3A) and in the number of SA‐β‐gal (+) cells (Figure 3B). Additionally, there is an upregulation of cyclin‐dependent kinase inhibitors p16^Ink4a^ and p15^Ink4b^ in cardiomyocytes, while expression of p21^CIP1^ remains unchanged when compared to young individuals (Figure 3C). The increased expression of the senescence biomarker p16^Ink4a^ in aging was validated by immunofluorescence staining in intact myocardium (Figure 3D). In addition, myocardial tissue from aged hearts exhibited a coordinated induction of SASP components spanning multiple functional categories, including pro‐inflammatory cytokines, immune cell recruitment signals, extracellular matrix remodeling and fibrotic mediators, as well as vasoactive and non‐canonical NF‐κB–linked factors. This transcriptional signature is consistent with the establishment of a complex, pro‐inflammatory and tissue‐modifying SASP in the aging myocardium (Figure 3E). Although precisely quantifying senescence is challenging since some senescence‐associated features are qualitative and there is no clear threshold defining its onset, quantification of SA‐β‐gal staining indicates that in the aging heart of otherwise healthy animals, 6%–8% of cardiomyocytes develop phenotypic changes consistent with proinflammatory senescence transformation.

### 3.4 Chronic Glycative Stress Causes Non‐Lethal Mitochondrial Damage and Lysosomal Dysfunction in H9c2 Cells

**FIGURE 4:**
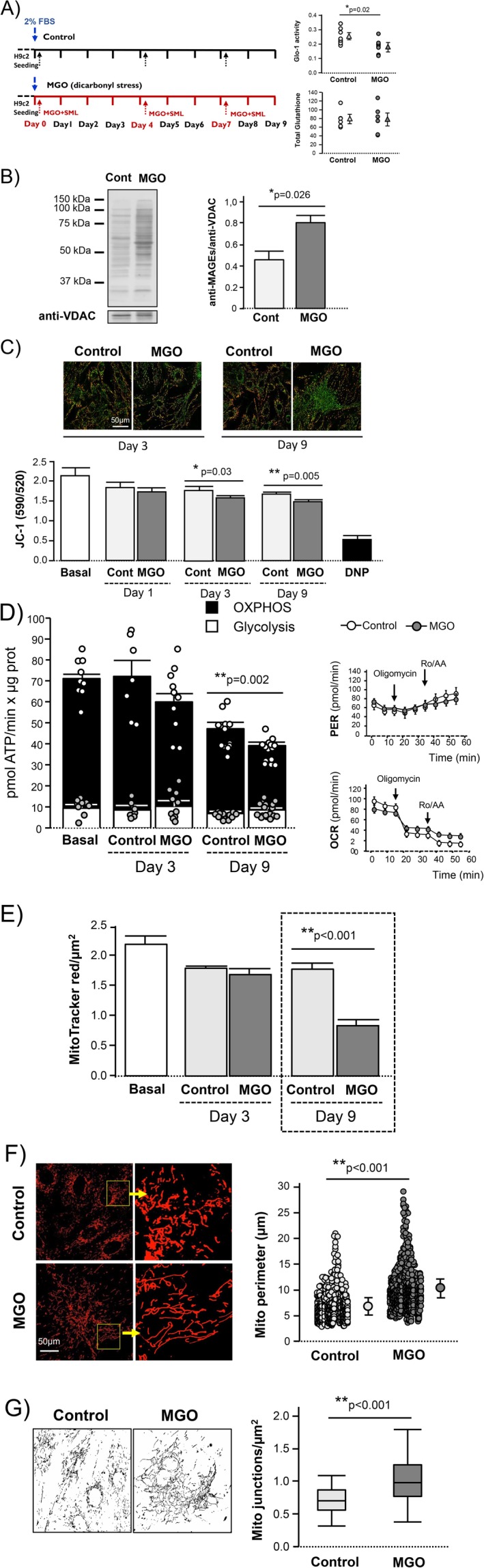
Glycative stress induces functional changes and structural remodeling in the mitochondria of H9c2 cells. (A) Protocol used to induce glycative stress in H9c2 cells. Dashed arrows indicate the time points at which serum‐starved medium (2% FBS) was renewed and MGO (80 μmol/L) + SML1306 (2 μmol/L) was added in the group of glycative stress. Cells from the control group were subjected to the same medium changes. Upper graph shows the quantification of Glo‐1 activity (nmols S‐lactoylglutathione/mg protein × min) and bottom graph represents total glutathione levels (nmols/mg of protein) after 24 h of induction of glycative stress, and the corresponding controls. Data correspond to mean ± SEM of *n* = 3 independent experiments. (B) Western blot of isolated mitochondria from H9c2 cells previously exposed to glycative stress (MGO) or control conditions for 9 days, immunolabeled against MGO‐derived AGEs (MAGEs). Bar graphs represent the quantification of the optical density (OD) of the total bands, expressed with respect to the loading control voltage dependent anion channel (VDAC). Data correspond to mean ± SEM of *n* = 3 independent experiments. (C) Confocal images (40×) of H9c2 cells labeled with JC‐1 as an indicator of ΔΨm, after 3 and 9 days of exposure to glycative stress (MGO) or control conditions (only the time points at which ΔΨm begins to decline in the MGO group relative to the control are shown). Bar graph represents the emission ratio of JC‐1 (590/520 nm) under basal conditions and at Days 1, 3, and 9 of glycative stress versus control. Maximal mitochondrial depolarization was achieved by the addition of DNP (200 μmol/L) and did not differ between treatment groups. Data correspond to mean ± SEM of *n* = 50–150 cells/group of 3 independent experiments. (D) Total ATP production rate (normalized to μg protein) in H9c2 cells under basal conditions and after 3 and 9 days of exposure to glycative stress (MGO) or control conditions. The stacked bars depict the relative contribution of glycolysis (white) and oxidative phosphorylation (black) to total ATP generation. The panels on the right show the oxygen consumption rate (OCR) and proton efflux rate (PER) after 9 days of exposure to glycative stress (MGO) or control conditions, as quantified by Seahorse. The arrowheads indicate the points at which oligomycin and rotenone/antimycin A (Rot/AA) were injected. Data correspond to mean ± SEM of *n* = 4 independent experiments. (E) Mitochondrial pool as quantified by MitoTracker Red (+) particles per μm^2^ in H9c2 cells at different time points after exposure to glycative stress (MGO) or control conditions (mean ± SEM, of 100–130 cells/group from *n* = 3 independent experiments). (F) Confocal images (40×) of H9c2 cells labeled with MitoTracker Red after 9 days of exposure to glycative stress (MGO) or control conditions (the yellow box is the area used to show an amplification of the optical field), and the corresponding quantification of mitochondrial perimeter in both groups (dot plots). Data correspond to mean ± SEM of *n* = 50–70 cells/group from 3 independent experiments. (G) Black and white images show skeletonized H9c2 cells obtained from MitoTracker Red labeling after 9 days of exposure to glycative stress (MGO) or control conditions. Box plots represent the degree of mitochondrial branching, expressed as the number of mitochondrial junctions per area (μm^2^) in *n* = 50–70 cells/group from 3 independent experiments.

To establish the causal role of glycative stress on aging‐associated cardiomyocyte remodeling described above, H9c2 cells were chronically exposed to low‐grade Glo‐1 pharmacological inhibition (SML1306) in the presence of MGO (Figure 4A). Partial inhibition of Glo‐1 was confirmed by the quantification of the rate of generation of S‐lactoylglutathione, a key intermediate in the Glo pathway, without changes in total glutathione (Figure 4A). On Day 9 of glycative stress, isolated mitochondria from H9c2 cells showed a significant increase in MAGEs as detected by western blot analysis (Figure 4B). Mitochondrial glycative damage was compatible with cell life and did not increase the rate of time‐dependent cell death (14.2% ± 2.1% after 9 days of glycative stress vs. 10.4% ± 2.7% in control conditions, *p* = ns). Glycative stress induced an early functional response in mitochondria, consisting of a slight but significant decrease in ΔΨm that did not recover throughout time, as detected by changes in DNP‐sensitive JC‐1 ratio fluorescence (Figure 4C), and a trend towards a reduction of mitochondrial ATP generation from oxidative phosphorylation without changes in the generation of ATP from glycolytic origin (Figure 4D), as compared to control cells. Unlike functional changes, which started to be apparent from early stages, the morphological remodeling of mitochondria in response to glycative stress appeared at later stage (Day 9), and included reduced cell mitochondrial density (Figure 4E), increased mitochondrial size with the appearance of “aging‐like” giant mitochondria (Figure 4F), and higher complexity of mitochondrial branching and interconnectivity with respect to control cells (Figure 4G). Importantly, the reduction in mitochondrial density observed in cells exposed to glycative stress appears to result from the cytosolic expansion of senescent cells outpacing the expansion of their mitochondrial compartment, as overall citrate synthase (CS) activity—a well‐established proxy for total mitochondrial mass—did not differ between control and glycated cells at Day 9 (0.65 ± 0.02 CSU/mg vs. 0.66 ± 0.03 CSU/mg, *p* = ns). In summary, mitochondria are one of the critical targets of non‐lethal glycative stress, and the resulting chemical damage alters their aerobic capacity and morphology in ways that resemble the mitochondrial changes observed in aging cardiomyocytes.

**FIGURE 5:**
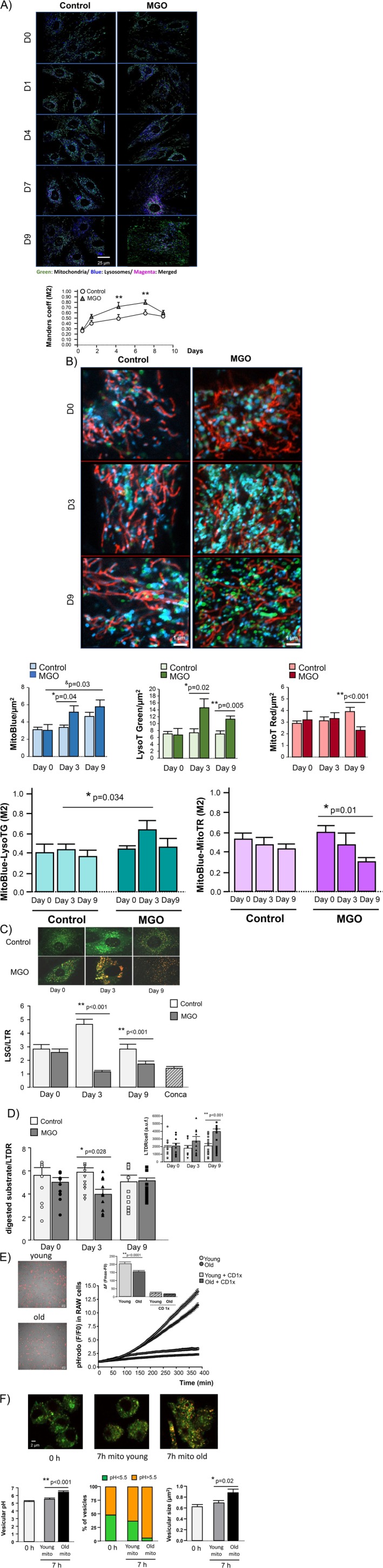
Glycative stress impairs lysosome digestion of defective mitochondria. (A) Lysosome‐mitochondria physical contacts throughout time (Days 0, 1, 4, 7 and 9) as quantified from confocal images (40×) of H9c2 cells exposed to glycative stress (MGO) and control conditions simultaneously labeled with MitoTracker Red (light green pseudocolor) and LysoTracker Green (dark blue pseudocolor), and the corresponding colocalizing pixels (magenta). The graph represents the time‐course of the Mander's overlap coefficient (colocalizing LysoTracker Green pixels with MitoTracker Red pixels with respect to total LysoTracker Green pixels, M2). Data correspond to mean ± SEM of *n* = 20–50 cells/group from 3 independent experiments. (B) Zoomed‐in confocal images of lysosome‐mitochondrial functional interaction throughout time (Days 0, 3, and 9) of H9c2 cells exposed to the same conditions as in A followed by a triple labeling to delineate mitochondria (MitoTracker Red), lysosomes (LysoTracker Green), and the functional inter‐organelle exchange (MitoBlue, which can be either in mitochondria or lysosomes depending on the mitophagy state). Colocalizing blue and red color (or close proximity between them) corresponds to mitochondria, with a slight gap in their overlay due to the high mitochondrial mobility of living cells. Colocalizing blue and green color (turquoise) or close proximity between them corresponds to autolysosomes containing mitochondrial material. Non‐colocalizing blue color corresponds to mitochondrial vesicles as part of the physiological quality control system. The concurrence of blue, red, and green (yellow) corresponds to mitophagy. Green color corresponds to resting lysosomes. Bar graphs represent the quantification of the different channels and the corresponding overlaps between them. Data are presented as mean ± SEM of *n* = 3 independent experiments. (C) Confocal images (60×) of H9c2 cells at different time points of exposure to glycative stress (MGO) or control conditions, labeled with pH‐sensitive LysoSensor Green (LSG) and pH‐insensitive LysoTracker Red (LTR). The reduction in LSG/LTR emission fluorescence corresponds to intralysosomal alkalinization. Positive control of lysosomal alkalinization was achieved by the addition of concanamycin A (10 nmol/L). Bar graph corresponds to mean ± SEM of *n* = 12–15 cells/group of 3 independent experiments. (D) Lysosomal hydrolytic activity (fluorescence emission at 90 min after the addition of the substrate) normalized by lysosomal pool (LysoTracker Deep Red, LTDR) in H9c2 cells exposed to glycative stress (MGO) or control conditions, as quantified by flow cytometry. The inset shows the variation in the total lysosomal pool per cell at different time points. The bar graphs correspond to mean ± SEM of *n* = 3 independent experiments. (E) Bright‐field images superimposed on fluorescent images (×20) of RAW 264.7 macrophages after 400 min of co‐incubation with pHrodo‐labeled mitochondria isolated from young and old mice. The graph depicts time‐dependent changes in pHrodo fluorescence, indicating the uptake (engulfment) and subsequent lysosomal degradation of mitochondria. The inset shows the cumulative increase in macrophage fluorescence over the experimental period following the exposure to mitochondria from young vs. old mice. Cytochalasin D (CD 1×) was applied to a subset of macrophages to inhibit phagocytosis. Data are presented as mean ± SEM and correspond to *n* = 100–120 cells/group of 3 independent experiments. (F) Confocal images (60×) of RAW 264.7 macrophages labeled with pH‐sensitive LysoSensor Green and pH‐insensitive LysoTracker Red at baseline (0 h) and after 7 h of exposure to cardiac mitochondria from young and old mice. Graphs show mean vesicular pH (left), percentage of acidic (pH < 5.5) and more alkaline (pH > 5.5) vesicles (middle), and mean vesicle size (right) at the indicated time points. Data are presented as mean ± SEM and correspond to *n* = 80–100 cells/group of 3 independent experiments.

We next investigated the impact of glycative stress on the activation of the lysosome‐mitochondria axis. Exposure of H9c2 cells to glycative stress prompted an early lysosome‐mitochondria interaction that remained elevated during the first 7 days and decreased thereafter, as detected by the degree of overlap between MitoTracker Red and LysoTracker Green (Figure 5A). To assess the mitochondrial functional interaction with lysosomes, we used a triple staining consisting of MitoTracker Red, LysoTracker Green, and MitoBlue. MitoBlue is a fluorescent bisamidine that initially distributes into functional mitochondria following the electrostatic gradient, but unlike other organelle‐specific dyes, it leaves the mitochondria in the form of vesicle‐like structures and eventually re‐localizes to lysosomes as part of the mitochondrial quality control (Sánchez et al. 2020). In H9c2 cells, glycative stress stimulated the migration of MitoBlue from mitochondria to lysosomes, as quantified by a higher degree of colocalization between LysoTracker Green and MitoBlue with respect to control conditions (Figure 5B). This increased interaction was statistically significant at Day 3, but it returned to baseline level after 9 days of glycative stress (Figure 5B). Despite the eventual normalization of inter‐organelle communication, glycative stress triggered a persistent increase in the lysosomal pool (LysoTracker Green staining, Figure 5B) and stimulated the formation of mitochondrial vesicles (MitoBlue staining, Figure 5B), indicating an accumulation of mitochondrial derived vesicles that are not efficiently engulfed by lysosomes to form autophagosomes (Sánchez et al. 2020). On the contrary, the amount of MitoBlue remaining inside the mitochondria tended to decrease over time in response to glycative stress (less colocalization between MitoTracker Red and MitoBlue, Figure 5B), and the mitochondrial density was eventually reduced with respect to control cells (MitoTracker Red staining, Figure 5B). These data indicate that despite the transient activation of mitophagy secondary to mitochondrial damage, lysosomes failed to eliminate defective mitochondria in response to glycative stress, and this failure was not paralleled by a compensatory increase in mitochondrial biogenesis.

Intraluminal acidic pH is essential for proper functioning of lysosomal hydrolases and consequently for the digestion of defective mitochondria. To investigate the effect of glycative stress on lysosomal pH, H9c2 cells were labeled with pH‐sensitive LysoSensor Green and counterstained with LysoTracker Red to delineate the lysosomal pool. Glycative stress induced an early and significant alkalinization of lysosomes (i.e., significant decrease of LysoSensor Green with respect to LysoTracker Red) in contrast to control cells that underwent a transient lysosomal acidification in response to the serum‐starvation of the culture medium (Figure 5C). The increase in lysosomal pH in response to glycative stress was not subsequently recovered and resembled that of the cells exposed to the V‐ATPase inhibitor concanamycin A (Figure 5C).

Lysosomal hydrolytic activity was quantified by flow cytometry in LysoTracker Deep Red‐loaded H9c2 cells added with a self‐quenched substrate that enters the cell by endocytosis and is de‐quenched upon its degradation by lysosomes. Lysosomal substrate digestion was then measured by the emitted fluorescent signal, which is proportional to the lysosomal activity and normalized by the total lysosomal pool. Exposure to glycative stress resulted in a significant reduction in the lysosomal hydrolytic activity of the cells at Day 3, which was subsequently restored (Figure 5D). Nevertheless, this apparent normalization of lysosomal hydrolytic function occurred at the expense of a significant up‐regulation of the lysosomal mass, as indicated by the increase in LysoTracker Deep Red fluorescence (Figure 5D). Consistent with early lysosomal dysfunction, glycative stress impaired LC3B processing in H9c2 cells. Specifically, glycative stress blunted the LC3B‐II increase relative to LC3B‐I in response to the lysosomal inhibitor concanamycin A at 48 h, indicating altered autophagic flux (Figure S2).

To confirm the impact of age‐related mitochondrial glycation on lysosomal function, we employed an ex vivo model of pHrodo‐labeled cardiac mitochondria isolated from young and old mice, co‐incubated with RAW 264.7 macrophages, a cell line known for its robust phagocytic activity and well‐characterized lysosomal system. The rate of mitochondrial uptake by macrophages (engulfment), assessed by the initial slope of fluorescence increase, was not influenced by the age of the mitochondrial donor (0.22 ± 0.01 Δ a.u.f./min for macrophages exposed to young mitochondria vs. 0.23 ± 0.01 Δ a.u.f./min for those exposed to old mitochondria, *p* = n.s; Figure 5E). However, following engulfment, lysosomal degradation efficiency varied with mitochondrial donor age. Specifically, macrophages exposed to mitochondria from aged mice exhibited a significantly lower fluorescence slope compared to those exposed to young mitochondria (0.55 ± 0.02 Δ a.u.f./min vs. 0.67 ± 0.03 Δ a.u.f./min, respectively, *p* = 0.006), and achieved a lower maximal fluorescence within the experimental time frame (Figure 5E), indicative of impaired acidification and reduced degradative efficiency. Consistently, direct assessment of lysosomal pH revealed that vesicles in macrophages exposed to aged mitochondria were less acidic after 7 h (Figure 5F), with a higher percentage of more alkaline vesicles compared to macrophages that had engulfed mitochondria from young donors (Figure 5F). In addition, these vesicles were significantly enlarged, suggesting altered maturation dynamics (Figure 5F).

In conclusion, interaction with glycated mitochondria is associated with impaired lysosomal acidification, increased vesicle size, and reduced degradative efficiency. Despite a compensatory increase in lysosomal mass, these changes are insufficient to support effective mitochondrial clearance, leading to the persistence of dysfunctional mitochondria and reduced mitochondrial density.

### 3.5 Glycative Stress Triggers Proinflammatory Cell Senescence

**FIGURE 6:**
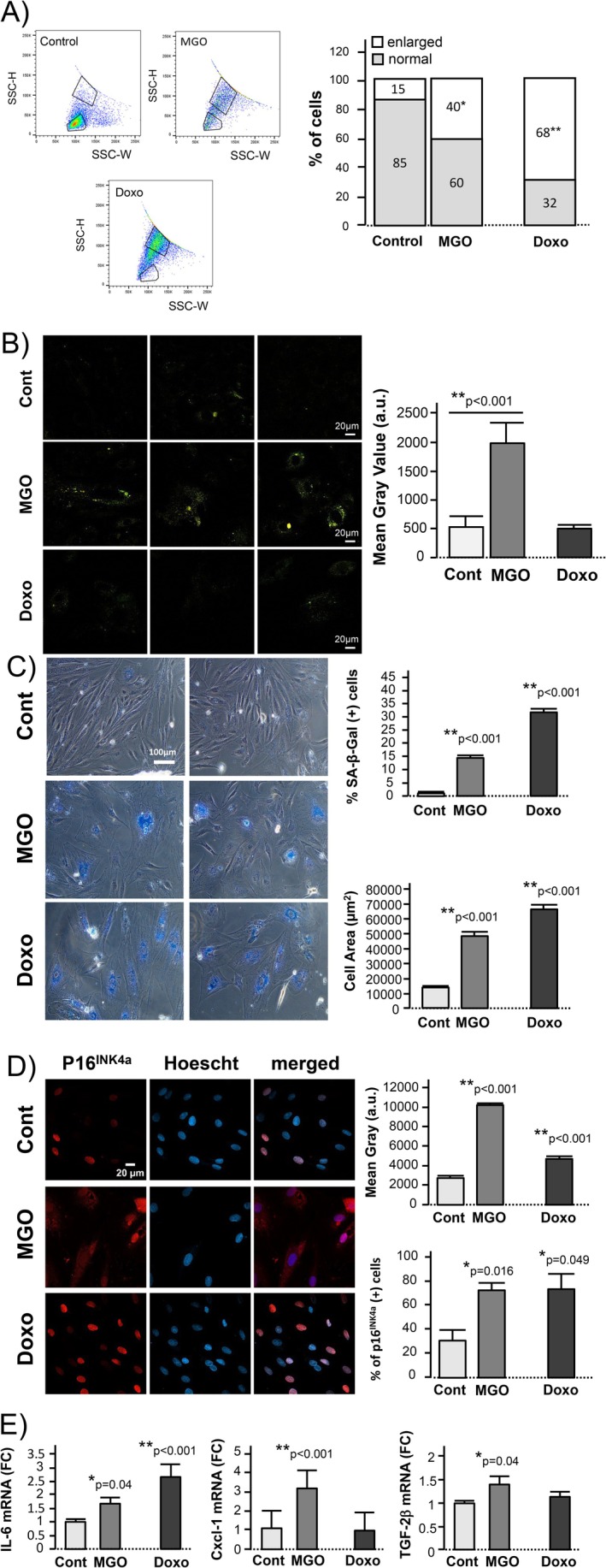
Exposure to low grade glycative stress triggers senescence in H9c2 cells. (A) Flow cytometry side‐scatter width (SSC‐W) versus side‐scatter height (SSC‐H) plots of single H9c2 cells obtained after 9 days of glycative stress or control conditions, in which normal sized‐cells (bottom window) and cells with abnormally enlarged morphology (upper window) were delimited. An additional group of cells treated with doxorubicin (Doxo) was used as a positive control of senescence‐induced cell enlargement. Right graph represents the percentage of normal cells and large cells in each treatment group. Data correspond to *n* = 3 independent experiments. *: *p* < 0.01 with respect to controls, **: *p* < 0.001 with respect to controls. (B) Confocal images (40×) of lipofuscin accumulation in H9c2 cells after 9 days of exposure to glycative stress (MGO), doxorubicin treatment (Doxo) or control conditions (3 examples per group). Bar graph (right) shows the quantification of total lipofuscin autofluorescence per cell. Data represent mean ± SEM of *n* = 10–12 cells/group of 3 independent experiments. (C) Bright field images of SA‐β‐galactosidase staining in H9c2 cells after 9 days of exposure to control, glycative stress (MGO), and doxorubicin (Doxo) treatment. The bar graphs represent the percentage of SA‐β‐galactosidase (+) cells (upper right) and the mean cell area (μm^2^) in each group (lower right). Data are presented as mean ± SEM of *n* = 2000–3000 cells/group from 4 independent experiments. (D) Confocal fluorescent images (40×) depicting p16^INK4a^ immunolabeling (red), Hoesch (blue nuclei) and the merged images in H9c2 cells after 9 days of exposure to the same treatments as in A. Bar graphs represent the quantification of the mean gray value per optical field (upper right) and the percentage of p16^INK4a^ (+) cells in each group (lower right). Data are presented as mean ± SEM of *n* = 300–400 cells/group from 3 independent experiments. (E) Expression levels of the SASP components interleukin‐6 (IL‐6), chemokine (C‐X‐C motif) ligand 1 (Cxcl‐1), and transforming growth factor 2b (TGF2b) in H9c2 cells after 9 days of exposure to the same conditions as in A, detected by RT‐PCR. The graphs represent the mean ± SEM of the fold change in gene expression calculated with respect to the control cells from *n* = 3 independent experiments (3 replicates per group).

Glycative stress induced additional morphological changes in H9c2 cells that started to be visible after 5–6 days and were significant after 9 days, including cell size enlargement (40% of cells developed a significant increase in size at Day 9, determined by flow cytometry discriminant analysis) and increased accumulation of lipofuscin pigment (Figure 6A,B). Because these changes may represent phenotypic traits of senescent transformation, we tested whether they can be recapitulated by the senescence inducer doxorubicin. While doxorubicin induced significant cell enlargement in 68% of cells (Figure 6A), it did not increase the lipofuscin content (Figure 6B), suggesting that the accumulation of indigestible material in response to glycative stress represents a specific fingerprint of glycative damage‐induced lysosomal dysfunction that more closely resembles the pathophysiology of senescence in aged cardiomyocytes. Other senescent markers, like the number of SA‐β‐gal (+) cells and of p16^Ink4a^ (+) cells, were significantly increased in H9c2 cells exposed to both glycative stress and doxorubicin compared with controls (Figure 6C,D). As in aging cardiomyocytes, senescence secondary to glycative stress also increased the expression of proinflammatory factors indicative of SASP development, in particular, the chemokine ligand Cxcl‐1 and the cytokine TFG‐β2 (Figure 6E). The effect of glycative stress on senescence induction was broadly similar to that observed with doxorubicin, although the time‐course and SASP profile differed between the two stressors. These data indicate that chronic exposure to glycative stress acts as an endogenous trigger of cell senescence.

## 4 Discussion

Although aging is the primary independent risk factor for the development of HFpEF, the biological mechanisms driving the transition from metabolically competent cardiomyocytes to dysfunctional cells remain poorly understood. In particular, the origins of the low‐grade, chronic inflammation characteristic of the aging heart—and its role in HFpEF onset and progression—are still unclear. In this study, we identify glycative stress, present in several aged tissues including the heart (Rabbani and Thornalley 2015; Ruiz‐Meana et al. 2019), as a significant metabolic contributor to mitochondrial proteostasis loss and impaired macroautophagy, ultimately triggering proinflammatory senescence in a subset of cardiomyocytes. Mass spectrometry revealed nine distinct advanced glycation end‐products (AGEs) that were significantly elevated in aged mouse hearts, with mitochondrial arginine residues being particularly susceptible to modification. Among the mitochondrial subpopulations, IFM were the most affected by aging‐associated glycative stress, while SSM remained relatively spared. Although mitochondrial glycation caused only mild bioenergetic impairment, it disrupted mitochondria‐lysosome communication (a key process in maintaining cellular homeostasis) and provoked lysosome alkalinization thereby reducing their proteolytic capacity. This conclusion is supported by the proof‐of‐concept experiments in cultured myoblasts exposed to glycative stress, where the lysosomal pH increased following the onset of mitophagy, and in macrophage‐like cells, where phagocytosis of mitochondria from aged mice impaired lysosomal digestion. Importantly, these experiments also revealed that vesicles containing aged mitochondria were significantly enlarged and more frequently alkaline, further supporting the notion that mitochondrial glycation alters lysosomal maturation and degradative function. The resulting lysosomal dysfunction led to the accumulation of undegradable material in the form of lipofuscin, an AGE‐rich polymer primarily derived from incompletely degraded mitochondria (Elleder et al. 1997). This cascade of events contributes to the persistence of damaged mitochondria and fuels the progression of maladaptive responses during aging, in part through the compensatory induction of cellular senescence.

### 4.1 Glycative Stress as a Catalyst of Cardiac Aging Through the Persistence of Dysfunctional Interfibrillar Mitochondria

Aerobic generation of ATP is so essential to cardiac function that heart mitochondria occupy up to 40% of cardiomyocyte volume, are more deeply invaginated (Brandt et al. 2017) and have evolved into specialized populations, that is, SSM and IFM, that differ not only in their spatial distribution but also in their biochemical composition, morphology, respiratory efficiency and calcium tolerance (Fernandez‐Sanz et al. 2014; Riva et al. 2005). IFM bear a higher metabolic burden, dynamically adjusting ATP production in response to contractile demands through tightly coupled calcium exchange with the sarcoplasmic reticulum (SR) (Eisner et al. 2013; Fernandez‐Sanz et al. 2014; Salazar‐Ramírez et al. 2021; Seidlmayer et al. 2019). Aging disproportionately affects IFM, leading to increased distancing between the SR and mitochondria (Fernandez‐Sanz et al. 2014), calcium phosphate precipitation in the mitochondrial matrix (Ruiz‐Meana et al. 2019), and a diminished ability of the cell to adapt to stress (Fernandez‐Sanz et al. 2014). In contrast, SSM are more susceptible to ischemic damage and diabetic cardiomyopathy (Hollander et al. 2014). Here, we show that mitochondria are the primary intracellular targets of glycative stress in the aging heart, with glycated protein accumulation preferentially affecting IFM. This selective mitochondrial damage correlates with the reduced aerobic function and fewer respiring IFM consistently observed in the aging heart in this and other studies (Lesnefsky et al. 2001). SSM, by contrast, appear structurally and functionally preserved. Although in cultured myoblasts mitochondrial organization is not similar to cardiomyocytes, exposure of these cells to glycative stress increased mitochondrial protein glycation, reduced ΔΨm and diminished aerobic capacity, precisely emulating features observed in aged cardiomyocytes. These functional traits were mild and compatible with cell life, but eventually led to mitochondrial remodeling, detected as an increased particle size and interconnectivity, and reduced mitochondrial density, indicative of impaired autophagy (Brunk and Terman 2002; Terman et al. 2003) and dysregulated stress response (Kim et al. 2020; Machiela et al. 2020). Analysis of AGEs composition reveals that they primarily arise from non‐enzymatic reactions between MGO or glyoxal and proteins (producing adducts such as CML, CMT [carboxymethyl‐tryptophan], MGH1 [MGO‐derived hydroimidazolone 1] and the MGO‐derived lysine dimer MDA54). These reactions are thermodynamically favored when the concentration of glucose‐derived dicarbonyl species is elevated, which is consistently observed in aging as a result of both impaired detoxification and increased production. On the one hand, the activity of Glo‐1, responsible for MGO catabolism into the non‐toxic D‐lactate, along with its cofactor glutathione, is significantly depressed in elderly humans and mice (Fernandez‐Sanz et al. 2014; Ruiz‐Meana et al. 2019). On the other hand, the age‐dependent increase in lipid peroxidation and amino acid catabolism (Miró et al. 2000; Timmerman and Volpi 2008) contributes to excessive generation of dicarbonyl compounds. Furthermore, since IFM provide the majority of cardiomyocyte ATP, their age‐dependent functional decline is likely to exacerbate MGO production. This effect may arise from impaired downstream glycolytic processing, resulting in the accumulation of the triose phosphate precursors glyceraldehyde‐3‐phosphate and dihydroxyacetone phosphate, which constitute the main endogenous source of MGO, even in the absence of increased glycolytic flux.

### 4.2 Impaired Ability of Lysosomes to Digest Mitochondrial AGEs Triggers Cell Senescence

Our study shows that in cardiomyocytes, aging is associated with an increase in lysosome number and size that, however, fails to protect mitochondria from the metabolic toxicity imposed by glycative stress, as evidenced by the persistence of glycated dysfunctional mitochondria. Lysosomes in aged cardiomyocytes are less acidic and some of them are lipofuscin‐loaded, a histological signature of age‐related neurodegenerative and cardiac diseases (Ahmed et al. 2010; Wellings et al. 2017). Exposure of myoblasts to low‐grade glycative stress mirrored lysosomal remodeling observed in aging, including a time‐dependent buildup of lipofuscin. It also increased the physical interaction between lysosomes and mitochondria, facilitating the cargo exchange, detected by inter‐organelle overlap and MitoBlue displacement. However, this response was transient and self‐limiting, likely due to lysosomal alkalinization and reduced proteolytic efficiency. The alkalinizing effect of mitochondrial AGEs on lysosomes was observed as a shift in LysoSensor Green fluorescence upon mitochondria‐lysosome interaction after 3 days of glycative stress and persisted over time, as in aged cardiomyocytes. It was accompanied by a reduction in cell hydrolytic efficiency, which was eventually compensated for by increased lysogenesis. Although the precise mechanism driving this lysosomal pH elevation remains unclear, our glycomic analyses excluded direct glycation of lysosomal V‐ATPase, and the moderate reduction in ATP observed under glycative stress is unlikely to fully account for impaired acidification. Instead, our findings point towards an indirect mechanism in which AGEs and AGE‐derived degradation products accumulate within lysosomes as non‐degradable material, disturbing intralysosomal homeostasis—through altered osmotic balance, reduced availability of essential cofactors, or impaired proton diffusion—ultimately contributing to lysosomal alkalinization. To confirm that lysosome alkalinization was due to the internalization of mitochondrial glycated proteins (and not to an indirect effect of MGO) macrophage‐like cells were exposed to cardiac mitochondria from young and aged mice. Phagocytosis of mitochondria from aged mice significantly impaired lysosomal acidification in macrophages, increased the proportion of more alkaline vesicles (pH > 5.5) and led to vesicle enlargement, supporting the notion that mitochondrial glycation directly alters lysosomal maturation and degradative function. The relevance of this mechanism in intact myocardium deserves further investigation.

We propose that impaired lysosomal clearance of glycated mitochondria plays a pivotal role in the geroconversion of cardiomyocytes during aging. Our data show that approximately 7% of cardiomyocytes in otherwise clinically asymptomatic aged mice, and 15% of myoblasts exposed to aging‐like glycative stress exhibit senescent traits, characterized by cell hypertrophy, β‐galactosidase positivity, increased expression of cyclin‐dependent kinase inhibitors and an elevated secretory profile (i.e., hyperfunctional state with increased expression of SASP). Importantly, whereas cell size enlargement was very pronounced in cultured myoblasts (which started to develop after 5–6 days of glycative stress and was very prevalent at Day 9), this type of morphological expansion is not feasible in the tightly packed cardiomyocytes of intact myocardium. Nonetheless, aged hearts showed a marked increase in cardiomyocyte cross‐sectional area, likely corresponding to a transitional state between adaptive and maladaptive (senescence‐induced) remodeling. Senescent cells contribute to chronic tissue inflammation and paracrine propagation of cell dysfunction by releasing pro‐inflammatory cytokines and extracellular matrix–remodeling enzymes (Balaraman et al. 2025; Hall et al. 2016). Consequently, their targeted elimination—via genetic approaches or pharmacological agents such as dasatinib, quercetin, navitoclax, and FOXO4‐DRI—has shown promise to improve cardiac function, reduce myocardial fibrosis and alleviate diastolic dysfunction in preclinical models of ischemia–reperfusion injury, chemotoxicity, aging and heart failure (Anderson et al. 2019; Baar et al. 2017; Dookun et al. 2020; Jia et al. 2020; Walaszczyk et al. 2019). In humans, although research is still emerging, elevated levels of senescence‐associated biomarkers and SASP components, such as p16^INK4a^, IL‐6 and TNF‐α, have been reported in individuals with HFpEF compared to healthy controls (Salman et al. 2024; Zhao et al. 2024), suggesting a mechanistic link between cellular senescence burden and disease progression.

**FIGURE 7:**
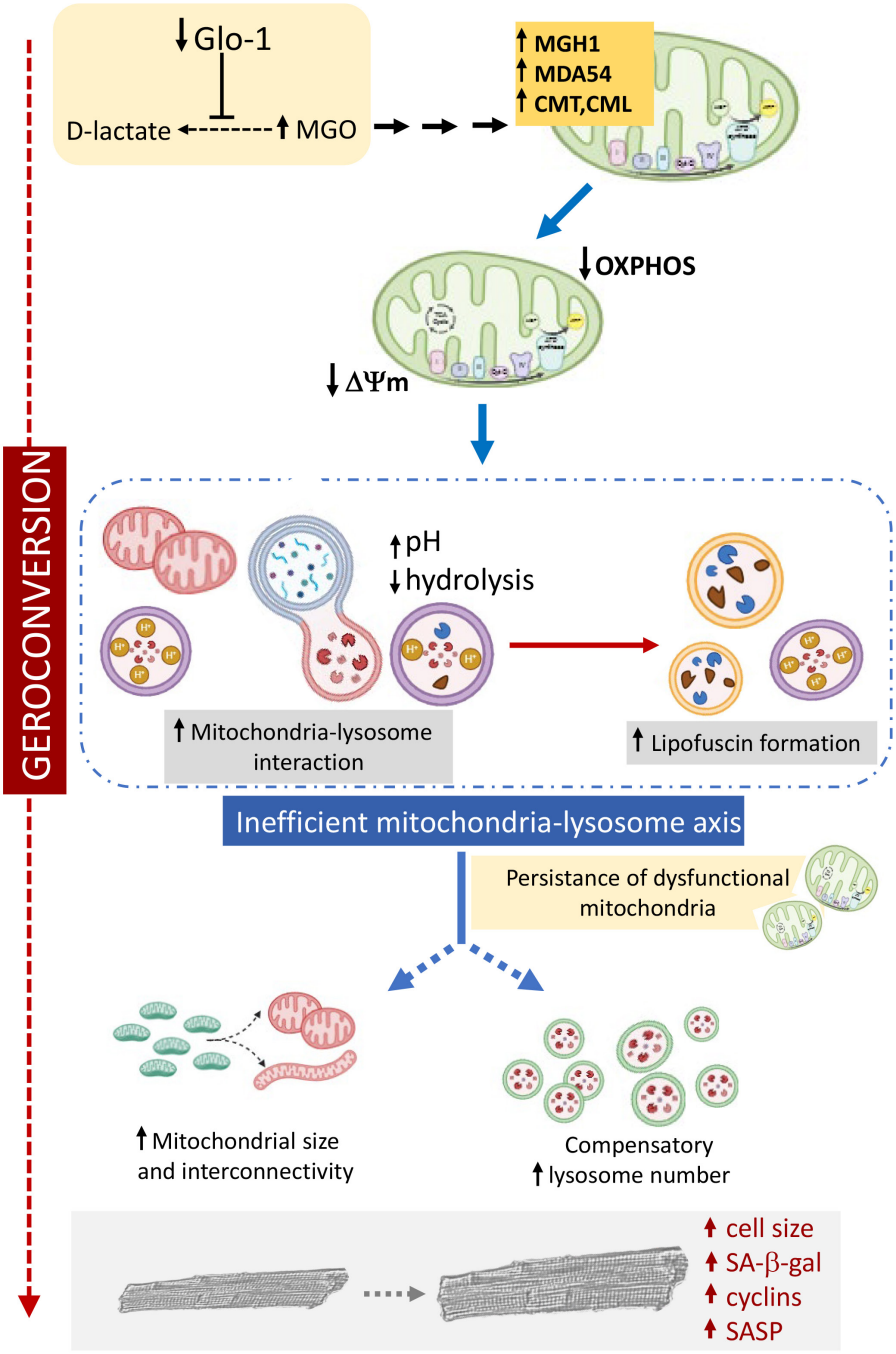
Proposed pathophysiological mechanism of geroconversion in cardiomyocytes during aging. Aging reduces glyoxalase‐1 (Glo‐1) activity, thermodynamically favoring the accumulation of advanced glycation end‐products (AGEs), which preferentially localize to mitochondria. The resulting mitochondrial glycative damage induces mild aerobic dysfunction, triggering the activation of the lysosomal response. However, mitochondria–lysosome crosstalk becomes inefficient due to lysosomal alkalinization following cargo exchange with mitochondria and reduced hydrolytic capacity, ultimately contributing to lipofuscin accumulation. The chronic persistence of unresolved mitochondrial damage drives structural remodeling of the mitochondrial network—characterized by increased size and interconnectivity—and promotes a compensatory expansion of lysosome number. Collectively, these alterations contribute to phenotypic transformation into a proinflammatory senescent state.

In conclusion, these data demonstrate the effect of glycative stress on cell senescence in vitro, suggesting a potential role of AGEs in driving cardiomyocyte senescence in old mice. Beyond this experimental evidence, our work places glycative stress within a broader physiological context in which the energetic demands of the heart exceed its homeostatic buffering capacity. The resulting intracellular damage caused by α‐oxoaldehyde–protein interactions outlines a pathophysiological sequence summarized in Figure 7. By integrating entropy‐based models of aging (Hayflick 1998) with the concept of aging as sustained overactivation of normal cellular processes (Blagosklonny 2022), the proposed mechanism provides a coherent framework and may enable more precise therapeutic strategies to protect the aging heart.

### 4.3 Study Limitations and Future Directions

Some limitations of the present study are that we focused exclusively on glucose‐related dicarbonyls, particularly MGO, and did not assess the potential contribution of highly reactive lipid‐derived dicarbonyls such as ONE and isoLGs, which may also influence cardiomyocyte senescence. Future work should determine the relative impact of these lipid species and their possible interaction with glycative stress. Furthermore, although our findings demonstrate an association between glycative stress and cardiomyocyte senescence, additional in vivo studies, and ultimately human data, will be required to substantiate a direct causal role of glycation products in cardiac aging.

## Author Contributions

Diana Bou‐Teen, Simonas Valiuska, Elisabet Miro‐Casas, and Chiara Rubeo performed experiments, collected and interpreted data, and carried out statistical analyses. Elena Bonzon‐Kulichenko and Jesús Vázquez conducted the proteomics and glycomics experiments and performed massive data analysis. Zuzana Nichtova and Celia Fernandez‐Sanz performed TEM and morphometric analysis of myocardium. Javier Inserte, Antonio Rodriguez‐Sinovas, Begoña Benito, and Eduard Ródenas‐Alesina contributed to data collection and interpretation from intact cardiac tissue. Marisol Ruiz‐Meana and Ignacio Ferreira‐González conceived and designed the study, supervised the experiments, and were responsible for data analysis, interpretation of results, and manuscript drafting. All authors contributed to manuscript revision and approved the final version.

## Funding

This study was funded by the Instituto de Salud Carlos III of the Spanish Ministry of Health through the projects FIS‐PI22‐00513, FIS PI23/00068, TACTICS (FORT23/0034, FORTALECE program), PID2021‐122348NB‐I00, S2022/BMD‐7333‐CM (INMUNOVAR‐CM) and by Generalitat de Catalunya (PERIS‐SLT028/23/195 and AGAUR 2021 SGR758), and co‐founded by the European Union (FEDER) and “La Caixa” Foundation under the project code LCF/PR/HR22/52420019.

## Conflicts of Interest

The authors declare no conflicts of interest.

## Supporting information


**Data S1:** acel70444‐sup‐0001‐supinfo.docx.

**Figure S1:** Altered mitochondrial cristae in the aging heart. High‐magnification TEM images of myocardium from 24‐month‐old mice, showing various examples of pathological mitochondrial cristae architecture. Panels illustrate different alterations, including semicircularly arranged cristae (red arrows), areas of low cristae density (yellow arrows) or with cristae loss (green arrow), and abnormally widened cristae tips (blue arrows), among others. These morphological changes are indicative of aging‐associated mitochondrial remodeling and are not observed in young mice.
**Figure S2:** Differential LC3B turnover in control versus glycative stress. Representative immunoblot and quantification of the LC3B‐II/LC3B‐I ratio in H9c2 cells exposed to control (Ctrl) or glycative stress (MGO) conditions for 48 h, in the presence or absence of the V‐ATPase inhibitor concanamycin A (ConA) (bar graph). Vinculin was used as a loading control. The LC3B‐II/LC3B‐I ratio increases in response to ConA in control cells (red arrow), consistent with active basal autophagic flux, whereas this response is attenuated under glycative stress (*p* = 0.136).

## Data Availability

Requests for further information and resources should be directed to and will be fulfilled by the lead contact, Marisol Ruiz‐Meana (mruizmeana@gmail.com). This study did not generate new unique reagents. Proteomics and glycomics data have been deposited in the PRIDE repository.
