# Supplementary material for: Glycative Stress Disrupts the Mitochondrial‐Lysosome Axis and Promotes Geroconversion in Aging Cardiomyocytes
Source: Aging Cell. 2026 Mar 13;25(3):e70444. doi: 10.1111/acel.70444 (PMC13093599; doi:10.1111/acel.70444)
Supplement: Supplementary file 1 — Data S1: acel70444‐sup‐0001‐supinfo.docx. [file ACEL-25-e70444-s002.docx]

**SUPPLEMENTAL METHODS**

**Mouse myocardium**

Mass spectrometry analysis, and modified peptide and protein identification and quantification

Protein extracts were obtained by mouse heart tissue homogenization with ceramic beads (MagNa Lyser Green Beads apparatus, Roche, Germany) in extraction buffer (50mmol/L Tris-HCl, 1mmol/L EDTA, 1.5% SDS, pH 8.5). Proteins were digested on-filter by the Fasilox approach using 50 mM iodoacetamide to block free (reduced) thiol groups and 40 mM DTT followed by 50 mM N-ethyl-maleimide to reduce-alkylate disulfide bonds (1). The resulting peptides were labeled with TMT10-plex following manufacturer instructions. The TMT10-plex experiment was composed by 4 biological replicates coming from young, 4 from old, and two channels reserved for internal standard (I.S.) samples. The I.S. was created by pooling all the samples and was used as reference to express relative quantification values. The labelled peptides were separated by high pH reversed-phase (Thermo Scientific) into 5 fractions and analyzed by nano-liquid chromatography-tandem mass spectrometry (nanoLC-MS/MS) using a FUSION mass spectrometer (Thermo Scientific). Peptide and protein identification were performed using the SEQUEST HT algorithm integrated in Proteome Discoverer 2.1 (Thermo Scientific). MS/MS scans were searched against a mouse target database (UniProtKB/Swiss-Prot, July 2016, 16958 protein sequences) including as variable modifications the following AGEs in 5 different search batches: 2-ammonio-6-[4-(hydroxymethyl)-3-oxidopyridinium-1-yl]-hexanoate (HMOP, 108.021129 Da) in Lys, hydroxyphenylglyoxal (1HPG, 132.021129 Da) in Arg, bis-hydroxphenylglyoxal (BHPG, 282.052824 Da) in Arg, dihydroxyimidazolidine in Arg (DHI, 72.021129 Da), glyoxal-derived hydroimiadazolone (G-H1, 39.994915 Da) in Arg, malondialdehyde adduct (MDA, 54.010565 Da) in Lys and Arg, and carboxymethyl (CM, 58.005479 Da) in Lys and Trp residues. Other variable modifications included Met oxidation (15.994915 Da), Cys carbamidomethylation (57.021464 Da) and methylthiolation (45.987721 Da), and TMT10-plex (229.162932 Da) on Lys and peptide N-terminus. Precursor mass tolerance was set to 800 ppm and fragment mass tolerance at 0.03 Da; precursor charge range was set to 2-4; and 3 was the maximum fragment charge. 2 miss-cleavages were allowed and only y- and b-ions were used for scoring. The same MS/MS spectra were also searched against an inverted database constructed from the same target database. False discovery rate (FDR) of peptide identifications was calculated by the refined method with an additional filter for precursor mass tolerance of 15 ppm (2). Quantitative information was extracted from MS/MS spectra of TMT-labeled peptides using Proteome Discoverer 2.1 (Thermo Scientific). Modified peptides and protein quantification were performed using the Generic Integration Algorithm (3) on the basis of the WSPP model (4) with some modifications. Peptide and protein abundance changes are expressed in standardized units corrected by the corresponding protein abundance (Zpq). Differences between the distributions of Zpq were analyzed by two-tailed Kolmogorov-Smirnov test.

**REFERENCES**

(1) [Elena Bonzon-Kulichenko](https://pubmed.ncbi.nlm.nih.gov/?sort=date&term=Bonzon-Kulichenko+E&cauthor_id=31874222), [Emilio Camafeita](https://pubmed.ncbi.nlm.nih.gov/?sort=date&term=Camafeita+E&cauthor_id=31874222), [Juan Antonio López](https://pubmed.ncbi.nlm.nih.gov/?sort=date&term=L%C3%B3pez+JA&cauthor_id=31874222), [María Gómez-Serrano](https://pubmed.ncbi.nlm.nih.gov/?sort=date&term=G%C3%B3mez-Serrano+M&cauthor_id=31874222), [Inmaculada Jorge](https://pubmed.ncbi.nlm.nih.gov/?sort=date&term=Jorge+I&cauthor_id=31874222), [Enrique Calvo](https://pubmed.ncbi.nlm.nih.gov/?sort=date&term=Calvo+E&cauthor_id=31874222), [Estefanía Núñez](https://pubmed.ncbi.nlm.nih.gov/?sort=date&term=N%C3%BA%C3%B1ez+E&cauthor_id=31874222), [Marco Trevisan-Herraz](https://pubmed.ncbi.nlm.nih.gov/?sort=date&term=Trevisan-Herraz+M&cauthor_id=31874222), [Navratan Bagwan](https://pubmed.ncbi.nlm.nih.gov/?sort=date&term=Bagwan+N&cauthor_id=31874222), [José Antonio Bárcena](https://pubmed.ncbi.nlm.nih.gov/?sort=date&term=B%C3%A1rcena+JA&cauthor_id=31874222), [Belén Peral](https://pubmed.ncbi.nlm.nih.gov/?sort=date&term=Peral+B&cauthor_id=31874222), [Jesús Vázquez](https://pubmed.ncbi.nlm.nih.gov/?sort=date&term=V%C3%A1zquez+J&cauthor_id=31874222). Improved integrative analysis of the thiol redox proteome using filter-aided sample preparation. J Proteomics 2020 Mar1:214:103624. doi: 10.1016/j.jprot.2019.103624.

(2) [Diana Bou-Teen](https://pubmed.ncbi.nlm.nih.gov/?sort=date&term=Bou-Teen+D&cauthor_id=35233924), [Celia Fernandez-Sanz](https://pubmed.ncbi.nlm.nih.gov/?sort=date&term=Fernandez-Sanz+C&cauthor_id=35233924), [Elisabet Miro-Casas](https://pubmed.ncbi.nlm.nih.gov/?sort=date&term=Miro-Casas+E&cauthor_id=35233924), [Zuzana Nichtova](https://pubmed.ncbi.nlm.nih.gov/?sort=date&term=Nichtova+Z&cauthor_id=35233924), [Elena Bonzon-Kulichenko](https://pubmed.ncbi.nlm.nih.gov/?sort=date&term=Bonzon-Kulichenko+E&cauthor_id=35233924), [Kelly Casós](https://pubmed.ncbi.nlm.nih.gov/?sort=date&term=Cas%C3%B3s+K&cauthor_id=35233924), [Javier Inserte](https://pubmed.ncbi.nlm.nih.gov/?sort=date&term=Inserte+J&cauthor_id=35233924), [Antonio Rodriguez-Sinovas](https://pubmed.ncbi.nlm.nih.gov/?sort=date&term=Rodriguez-Sinovas+A&cauthor_id=35233924), [Begoña Benito](https://pubmed.ncbi.nlm.nih.gov/?sort=date&term=Benito+B&cauthor_id=35233924), [Shey-Shing Sheu](https://pubmed.ncbi.nlm.nih.gov/?sort=date&term=Sheu+SS&cauthor_id=35233924), [Jesús Vázquez](https://pubmed.ncbi.nlm.nih.gov/?sort=date&term=V%C3%A1zquez+J&cauthor_id=35233924), [Ignacio Ferreira-González](https://pubmed.ncbi.nlm.nih.gov/?sort=date&term=Ferreira-Gonz%C3%A1lez+I&cauthor_id=35233924), [Marisol Ruiz-Meana](https://pubmed.ncbi.nlm.nih.gov/?sort=date&term=Ruiz-Meana+M&cauthor_id=35233924). Defective dimerization of FoF1-ATP synthase secondary to glycation favors mitochondrial energy deficiency in cardiomyocytes during aging. Aging Cell 2022 Mar;21(3):e13564.

doi: 10.1111/acel.13564.

(3) [Fernando García-Marqués](https://pubmed.ncbi.nlm.nih.gov/?sort=date&term=Garc%C3%ADa-Marqu%C3%A9s+F&cauthor_id=26893027), [Marco Trevisan-Herraz](https://pubmed.ncbi.nlm.nih.gov/?sort=date&term=Trevisan-Herraz+M&cauthor_id=26893027), [Sara Martínez-Martínez](https://pubmed.ncbi.nlm.nih.gov/?sort=date&term=Mart%C3%ADnez-Mart%C3%ADnez+S&cauthor_id=26893027), [Emilio Camafeita](https://pubmed.ncbi.nlm.nih.gov/?sort=date&term=Camafeita+E&cauthor_id=26893027), [Inmaculada Jorge](https://pubmed.ncbi.nlm.nih.gov/?sort=date&term=Jorge+I&cauthor_id=26893027), [Juan Antonio Lopez](https://pubmed.ncbi.nlm.nih.gov/?sort=date&term=Lopez+JA&cauthor_id=26893027), [Nerea Méndez-Barbero](https://pubmed.ncbi.nlm.nih.gov/?sort=date&term=M%C3%A9ndez-Barbero+N&cauthor_id=26893027), [Simón Méndez-Ferrer](https://pubmed.ncbi.nlm.nih.gov/?sort=date&term=M%C3%A9ndez-Ferrer+S&cauthor_id=26893027), [Miguel Angel Del Pozo](https://pubmed.ncbi.nlm.nih.gov/?sort=date&term=Del+Pozo+MA&cauthor_id=26893027), [Borja Ibáñez](https://pubmed.ncbi.nlm.nih.gov/?sort=date&term=Ib%C3%A1%C3%B1ez+B&cauthor_id=26893027), [Vicente Andrés](https://pubmed.ncbi.nlm.nih.gov/?sort=date&term=Andr%C3%A9s+V&cauthor_id=26893027), [Francisco Sánchez-Madrid](https://pubmed.ncbi.nlm.nih.gov/?sort=date&term=S%C3%A1nchez-Madrid+F&cauthor_id=26893027), [Juan Miguel Redondo](https://pubmed.ncbi.nlm.nih.gov/?sort=date&term=Redondo+JM&cauthor_id=26893027), [Elena Bonzon-Kulichenko](https://pubmed.ncbi.nlm.nih.gov/?sort=date&term=Bonzon-Kulichenko+E&cauthor_id=26893027), [Jesús Vázquez](https://pubmed.ncbi.nlm.nih.gov/?sort=date&term=V%C3%A1zquez+J&cauthor_id=26893027). A Novel Systems-Biology Algorithm for the Analysis of Coordinated Protein Responses Using Quantitative Proteomics. Mol Cell Proteomics 2016 May;15(5):1740-60. doi: 10.1074/mcp.M115.055905.

(4) [Pedro Navarro](https://pubmed.ncbi.nlm.nih.gov/?sort=date&term=Navarro+P&cauthor_id=24512137), [Marco Trevisan-Herraz](https://pubmed.ncbi.nlm.nih.gov/?sort=date&term=Trevisan-Herraz+M&cauthor_id=24512137), [Elena Bonzon-Kulichenko](https://pubmed.ncbi.nlm.nih.gov/?sort=date&term=Bonzon-Kulichenko+E&cauthor_id=24512137), [Estefanía Núñez](https://pubmed.ncbi.nlm.nih.gov/?sort=date&term=N%C3%BA%C3%B1ez+E&cauthor_id=24512137), [Pablo Martínez-Acedo](https://pubmed.ncbi.nlm.nih.gov/?sort=date&term=Mart%C3%ADnez-Acedo+P&cauthor_id=24512137), [Daniel Pérez-Hernández](https://pubmed.ncbi.nlm.nih.gov/?sort=date&term=P%C3%A9rez-Hern%C3%A1ndez+D&cauthor_id=24512137), [Inmaculada Jorge](https://pubmed.ncbi.nlm.nih.gov/?sort=date&term=Jorge+I&cauthor_id=24512137), [Raquel Mesa](https://pubmed.ncbi.nlm.nih.gov/?sort=date&term=Mesa+R&cauthor_id=24512137), [Enrique Calvo](https://pubmed.ncbi.nlm.nih.gov/?sort=date&term=Calvo+E&cauthor_id=24512137), [Montserrat Carrascal](https://pubmed.ncbi.nlm.nih.gov/?sort=date&term=Carrascal+M&cauthor_id=24512137), [María Luisa Hernáez](https://pubmed.ncbi.nlm.nih.gov/?sort=date&term=Hern%C3%A1ez+ML&cauthor_id=24512137), [Fernando García](https://pubmed.ncbi.nlm.nih.gov/?sort=date&term=Garc%C3%ADa+F&cauthor_id=24512137), [José Antonio Bárcena](https://pubmed.ncbi.nlm.nih.gov/?sort=date&term=B%C3%A1rcena+JA&cauthor_id=24512137), [Keith Ashman](https://pubmed.ncbi.nlm.nih.gov/?sort=date&term=Ashman+K&cauthor_id=24512137), [Joaquín Abian](https://pubmed.ncbi.nlm.nih.gov/?sort=date&term=Abian+J&cauthor_id=24512137), [Concha Gil](https://pubmed.ncbi.nlm.nih.gov/?sort=date&term=Gil+C&cauthor_id=24512137), [Juan Miguel Redondo](https://pubmed.ncbi.nlm.nih.gov/?sort=date&term=Redondo+JM&cauthor_id=24512137), [Jesús Vázquez](https://pubmed.ncbi.nlm.nih.gov/?sort=date&term=V%C3%A1zquez+J&cauthor_id=24512137). J General statistical framework for quantitative proteomics by stable isotope labeling. Proteome Res 2014 Mar 7;13(3):1234-47. doi: 10.1021/pr4006958.
