# Supplementary material for: Glycative Stress Disrupts the Mitochondrial‐Lysosome Axis and Promotes Geroconversion in Aging Cardiomyocytes
Source: Aging Cell. 2026 Mar 13;25(3):e70444. doi: 10.1111/acel.70444 (PMC13093599; doi:10.1111/acel.70444)
Supplement: Supplementary file 2 — Figure S1: Altered mitochondrial cristae in the aging heart. High‐magnification TEM images of myocardium from 24‐month‐old mice, showing various examples of pathological mitochondrial cristae architecture. Panels illustrate different alterations, including semicircularly arranged cristae (red arrows), areas of low cristae density (yellow arrows) or with cristae loss (green arrow), and abnormally widened cristae tips (blue arrows), among others. These morphological changes are indicative of aging‐associated mitochondrial remodeling and are not observed in young mice. Figure S2: Differential LC3B turnover in control versus glycative stress. Representative immunoblot and quantification of the LC3B‐II/LC3B‐I ratio in H9c2 cells exposed to control (Ctrl) or glycative stress (MGO) conditions for 48 h, in the presence or absence of the V‐ATPase inhibitor concanamycin A (ConA) (bar graph). Vinculin was used as a loading control. The LC3B‐II/LC3B‐I ratio increases in response to ConA in control cells (red arrow), consistent with active basal autophagic flux, whereas this response is attenuated under glycative stress (p = 0.136). [file ACEL-25-e70444-s001.pptx]

## Slide 1
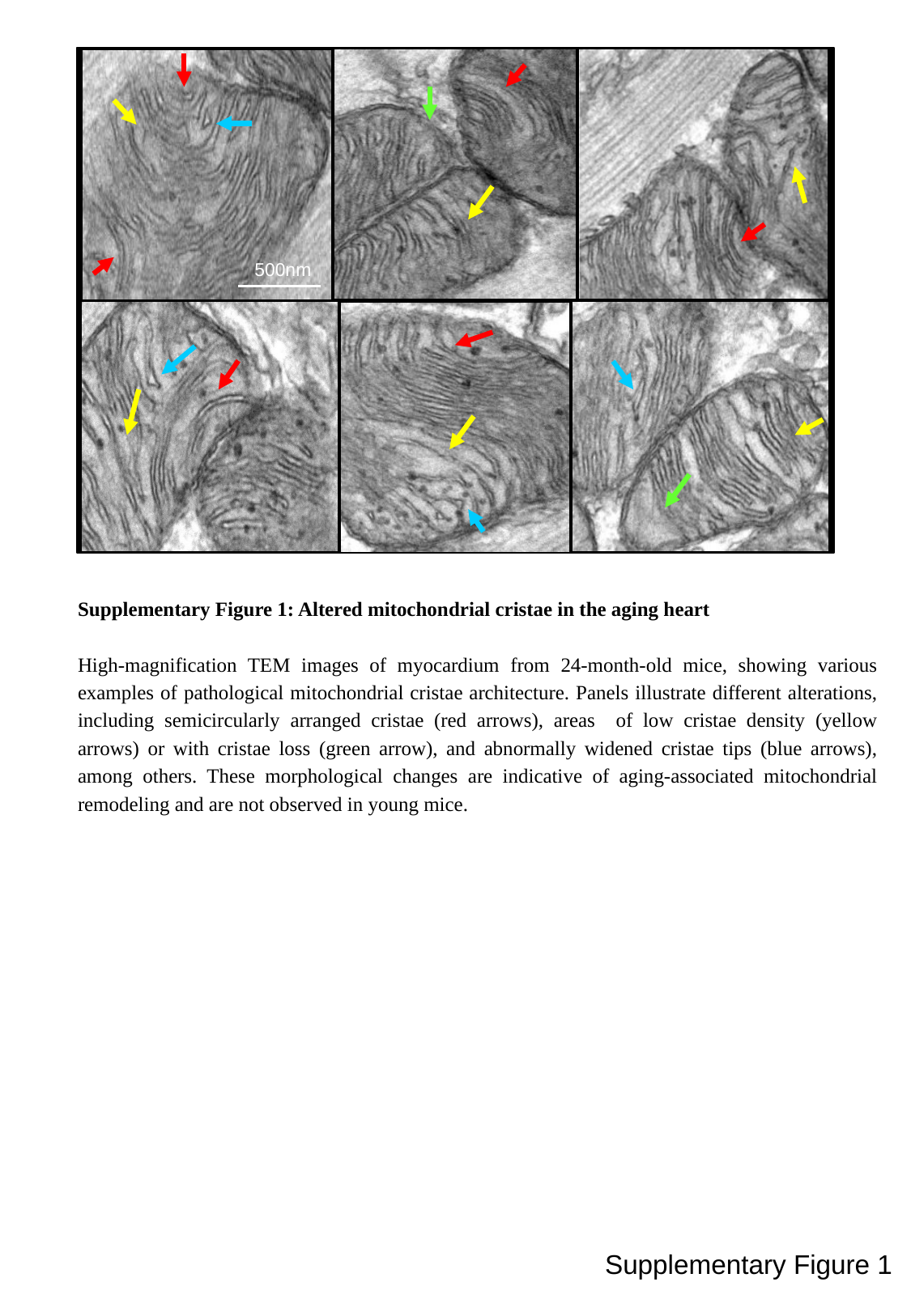

500nm
Supplementary Figure 1: Altered mitochondrial cristae in the aging heart
High-magnification TEM images of myocardium from 24-month-old mice, showing various examples of pathological mitochondrial cristae architecture. Panels illustrate different alterations, including semicircularly arranged cristae (red arrows), areas of low cristae density (yellow arrows) or with cristae loss (green arrow), and abnormally widened cristae tips (blue arrows), among others. These morphological changes are indicative of aging-associated mitochondrial remodeling and are not observed in young mice.
Supplementary Figure 1

## Slide 2
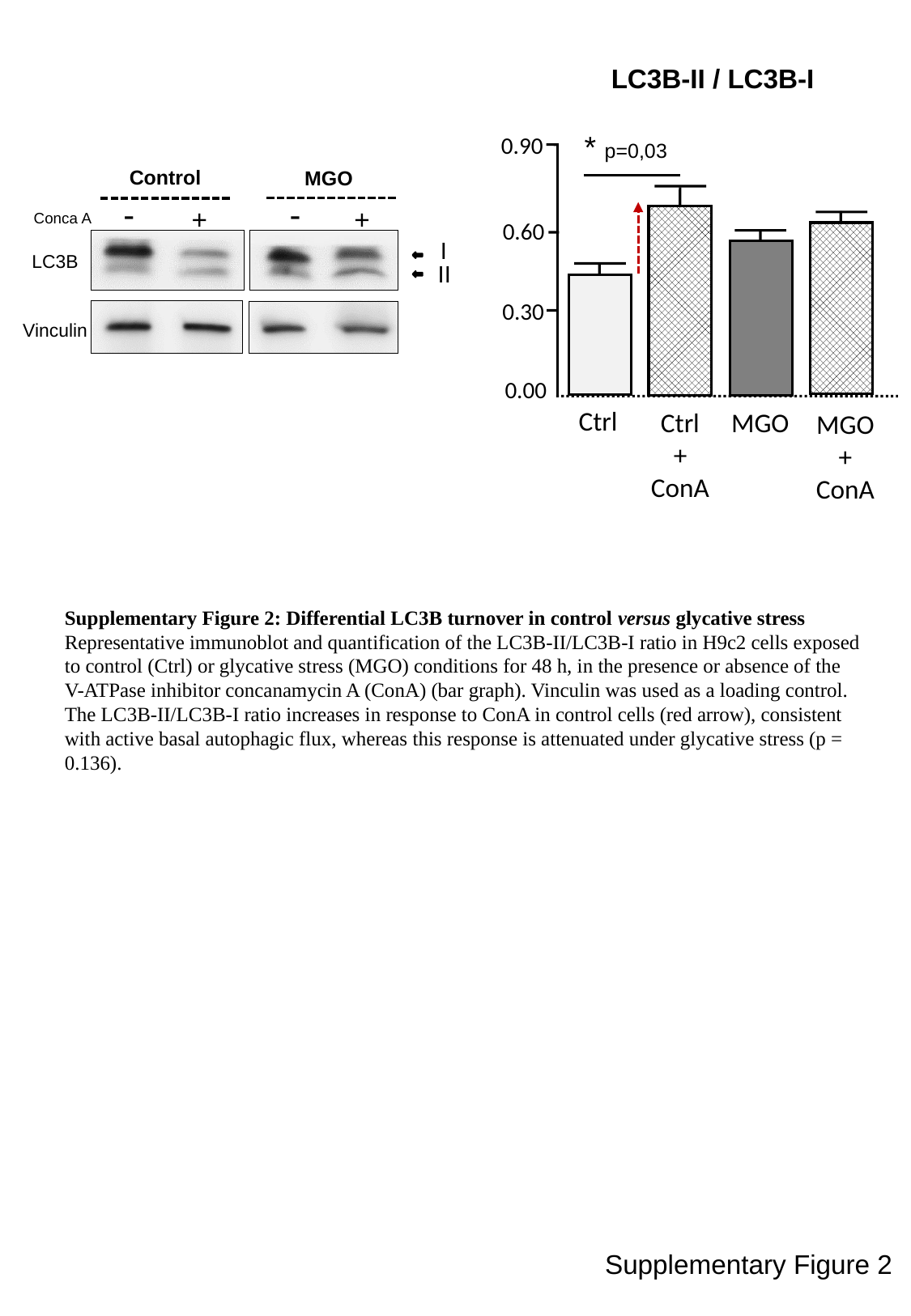

LC3B-II / LC3B-I
* p=0,03
0.90
Control
MGO
-
-
+
+
Conca A
I
LC3B
II
Vinculin
0.60
0.30
0.00
Ctrl
MGO
Ctrl
+
ConA
MGO
+
ConA
Supplementary Figure 2: Differential LC3B turnover in control versus glycative stress
Representative immunoblot and quantification of the LC3B-II/LC3B-I ratio in H9c2 cells exposed to control (Ctrl) or glycative stress (MGO) conditions for 48 h, in the presence or absence of the V-ATPase inhibitor concanamycin A (ConA) (bar graph). Vinculin was used as a loading control. The LC3B-II/LC3B-I ratio increases in response to ConA in control cells (red arrow), consistent with active basal autophagic flux, whereas this response is attenuated under glycative stress (p = 0.136).
Supplementary Figure 2
